# Genetic Analysis of Collective Motility of *Paenibacillus* sp. NAIST15-1

**DOI:** 10.1371/journal.pgen.1006387

**Published:** 2016-10-20

**Authors:** Kazuo Kobayashi, Yu Kanesaki, Hirofumi Yoshikawa

**Affiliations:** 1 Graduate School of Biological Sciences, Nara Institute of Science & Technology, Ikoma, Japan; 2 NODAI Genome Research Center, Tokyo University of Agriculture, Setagaya-ku, Japan; 3 Department of Bioscience, Tokyo University of Agriculture, Setagaya-ku, Japan; Indiana University, UNITED STATES

## Abstract

Bacteria have developed various motility mechanisms to adapt to a variety of solid surfaces. A rhizosphere isolate, *Paenibacillus* sp. NAIST15-1, exhibited unusual motility behavior. When spotted onto 1.5% agar media, *Paenibacillus* sp. formed many colonies, each of which moved around actively at a speed of 3.6 μm/sec. As their density increased, each moving colony began to spiral, finally forming a static round colony. Despite its unusual motility behavior, draft genome sequencing revealed that both the composition and organization of flagellar genes in *Paenibacillus* sp. were very similar to those in *Bacillus subtilis*. Disruption of flagellar genes and flagellar stator operons resulted in loss of motility. *Paenibacillus* sp. showed increased transcription of flagellar genes and hyperflagellation on hard agar media. Thus, increased flagella and their rotation drive *Paenibacillus* sp. motility. We also identified a large extracellular protein, CmoA, which is conserved only in several *Paenibacillus* and related species. A *cmoA* mutant could neither form moving colonies nor move on hard agar media; however, motility was restored by exogenous CmoA. CmoA was located around cells and enveloped cell clusters. Comparison of cellular behavior between the wild type and *cmoA* mutant indicated that extracellular CmoA is involved in drawing water out of agar media and/or smoothing the cell surface interface. This function of CmoA probably enables *Paenibacillus* sp. to move on hard agar media.

## Author Summary

Motility is crucial as it enables bacteria to seek out and colonize suitable environments. Here, a new isolate from the rhizosphere, *Paenibacillus* sp., exhibited unusual motility behavior. When spotted on low wet, hard agar media, the bacterium formed many colonies, each of which moved around actively like an individual organism. The cells in moving colonies had a large number of flagella, which drove colony movement. Newly identified large extracellular protein was essential to form moving colonies on hard agar media. This protein seems to facilitate motility by drawing water out of agar or smoothing the cell surface interface. On encountering a wet environment, the moving colonies disassembled quickly, and individual cells swam in the water layer, suggesting that moving colonies specially form under low wet conditions. The results describe a novel mechanism that explains how *Paenibacillus* sp. overcomes environmental challenges by moving on solid surfaces.

## Introduction

Migration is a critical mechanism by which bacteria survive and thrive in a particular environment. Motility enables bacteria to search for nutrients, avoid toxic compounds, and seek out favorable environmental niches that they can then colonize. The organelles responsible for mobility, flagella, are common in bacteria [1]. The most common form of flagella-dependent motility, called swimming motility, only works in an aqueous environment. However, bacteria live not only in aqueous environments but also on a variety of biotic and abiotic solid surfaces. Therefore, many bacteria have developed mechanisms that facilitate movement on a solid surface. These include swarming, twitching, gliding, and sliding motility, which are mediated by flagella, Type IV pili, focal adhesion complexes, surface active molecules, or the expansive forces generated by growing cells [2–4].

Swarming motility is defined as flagella-driven “group” movement across a solid surface [3, 5, 6], and is observed in several bacterial families [5]. Swarming motility is clearly distinct from swimming motility, which is the flagella-driven movement of “individual cells” in an aqueous environment. Indeed, under laboratory conditions, swarming motility is usually observed in solid media containing agar at concentrations above 0.5%, whereas swimming motility is observed in liquid or solid media containing agar at 0.3% or lower [2,5]. Since the motion of flagella pushes the cell forward against the surrounding water, surface water is a critical element for swarming motility as well as for swimming motility. However, water in hard agar media is usually trapped within the agar matrix. To overcome this, swarmer cells attract water to the surface from the agar matrix [7–9]. A high cell density, cellular secretions, and flagella rotation help to attract water to the surface [7–11]. Swarming motility requires differentiation into specialized cells, which often exhibit hyperflagellation, cell elongation, and the secretion of wetting agents that attract water or reduce surface tension. Swarming cells actively move in a fluid layer within swarm colonies, and often form small moving groups, called rafts, in which the cells closely aligned along their long axis [3, 5, 6]. The formation of rafts facilitates movement on hard agar media partly by reducing viscosity/drag on individuals [12], but its mechanism and function are still unclear. Since rafts are unstable and frequently change their members and shape, no substance or matrix appear to maintain rafts [5, 6].

*Paenibacillus* is a genus of facultative anaerobic, Gram-positive, spore-forming bacteria that are closely related to the genus *Bacillus* [13]. *Paenibacillus* spp. are isolated from various environments, and are often associated with plants [14–18]. Many strains of *Paenibacillus* spp. stimulate plant growth; for example, by assisting nutrient acquisition through nitrogen fixation and by producing cytokinins, peptide antibiotics, and volatiles that elicit a defense response [15, 19–22]. Certain species of *Paenibacillus*, such as *P*. *vortex*, *P*. *alvei*, and *P*. *dendritiformis* exhibit motility that is sufficiently robust to enable movement across the surface of media containing >1.5% agar [23–25]. Although the ecological role of *Paenibacillus* bacteria is largely unknown, their strong motility ability probably provides considerable advantages with respect to colonization of their natural habitats. Moreover, as *P*. *vortex* also facilitates the spread of other non-motile microbes and fungi that bind to *P*. *vortex* colonies [26, 27], robust motile *Paenibacillus* bacteria probably contributes to the dispersal of other microbes in their habitats.

Colonies formed by robust motile *Paenibacillus* bacteria form intricate patterns on agar media. *P*. *vortex* forms a highly branched colony pattern on agar media [24]. When grown on hard agar media, hundreds to millions of *P*. *vortex* cells assemble and generate rotating colonies (also referred to as wandering colonies). These rotating colonies move forward, leaving behind trails of cells; the latter form the ‘branches’ of the colony pattern. *P*. *alvei* forms a nebula colony pattern on agar plates, which comprises randomly scattered clusters of bacteria [23]. Although its colony pattern is quite different from that of *P*. *vortex*, *P*. *alvei* also forms wandering colonies [3], indicating that *P*. *vortex* and *P alvei* share common mechanisms for motility. *P*. *dendritiformis* forms a chiral branching colony pattern, where cells are aligned parallel to their neighbors [25]. At the tips of the growing branches, each cell moves back and forth along their neighbor [28]. Since these cells do not form wandering colonies, the mechanism underlying the motility of *P*. *dendritiformis* appears to be different from those of *P*. *vortex* and *P*. *alvei*. The molecular mechanisms underlying motility and colony pattern formation are very interesting but are unclear because these *Paenibacillus* bacteria are not amenable to genetic manipulation. For instance, although flagella are thought to produce the driving force, this remains unproven.

Here, we describe the motility behavior of a genetically tractable isolate of *Paenibacillus* sp. NAIST15-1 and its genetic analysis. *Paenibacillus* sp. showed peritrichous hyperflagellation in response to growth on hard agar media. Hyperflagellated cells formed moving colonies that were able to migrate across the surface of solid media containing >1.5% agar. We found that the *cmoA* gene (colony movement A) was necessary for motility on hard agar media. CmoA is a large extracellular protein that envelops moving cells clusters. CmoA is conserved in *Paenibacillus* bacteria that form moving colonies. The *cmoA* mutant were unable to move on 1.5% agar media but able to spread on 0.6% agar media. However, its cellular behavior on 0.6% agar media was quite different from that of the wild-type strain. We discuss possible roles of CmoA in motility on hard agar media.

## Results

### Motile behavior of *Paenibacillus* sp.

**Fig 1:**
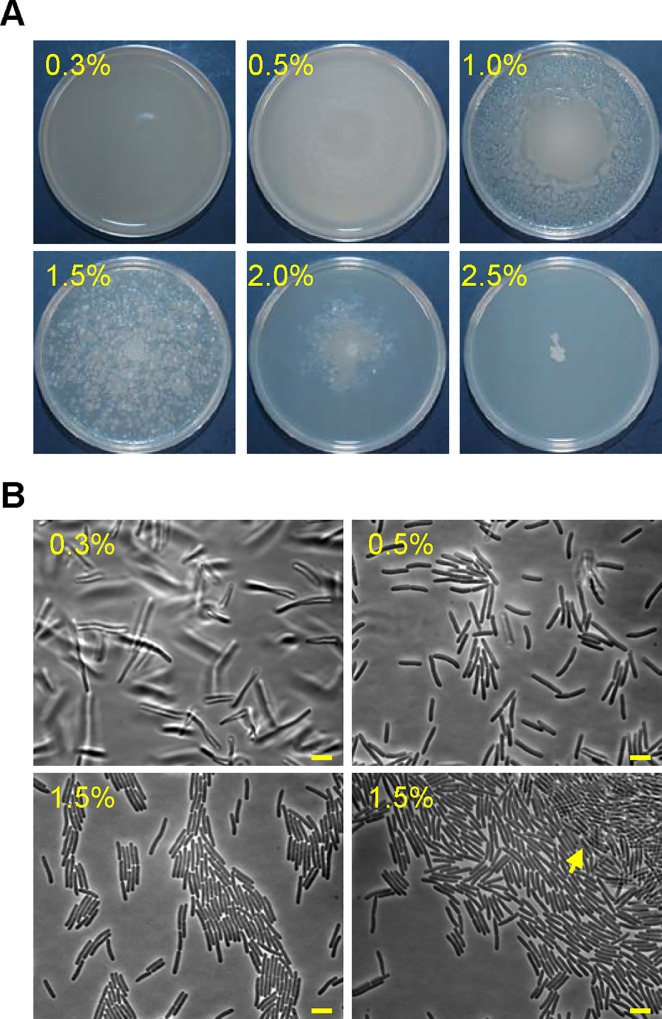
Motility of *Paenibacillus* sp. (A) Colony spreading pattern of *Paenibacillus* sp. The wild-type strain was inoculated onto the center of 2×YT plates containing a variety of agar concentrations and grown at 37°C for 18 h. Plate diameter, 9 cm. (B) Morphology of motile cells. The wild-type strain was inoculated onto the center of plates containing 0.3%, 0.5%, or 1.5% agar and incubated at 37°C for 6 h. Coverslips were placed directly on the surface of the leading edge zones of the colonies and cell morphology observed under a light microscope. Stacks of cells can be seen in the lower-right panel, and are indicated by an arrow. Scale bar, 5 μm.

*Paenibacillus* sp. NAIST15-1 (hereafter referred to as *Paenibacillus* sp.) was isolated from spore-forming bacteria that showed antagonistic activity against a plant pathogen *Fusarium oxyosporum* from weed roots and associated soil in the course of the previous study [29]. The bacterium produced swollen sporangia (S1 Fig), which is typical of the genus *Paenibacillus* [13]. Consistent with this, the 16S rDNA sequence of this bacterium was quite similar to those of *Paenibacillus* bacteria (S2 Fig); it showed 99% identity with that of *Paenibacillus alvei*. These observations indicated that the bacterium belonged to the genus *Paenibacillus*. *Paenibacillus* sp. exhibited unusual colony formation. When inoculated onto the center of plates containing 2×YT/1.5% agar media, *Paenibacillus* sp. spread over the surface and formed many discrete colonies (Fig 1A). This colony scattering phenotype appears similar as that observed for *Paenibacillus alvei*, and is described as either “wandering colonies” or “nebula pattern formation” [3, 23]. We were interested in this phenotype and a molecular mechanism behind it. Our isolate *Paenibacillus* sp. was suitable for the analysis because it was amenable to genetic manipulation (Methods, see below). The colony spreading pattern was greatly affected by the agar concentration (Fig 1A). *Paenibacillus* sp. formed a featureless mat all over plates containing 0.3% or 0.5% agar, but formed a mat within the central portion of 1% agar plates, with multiple colonies around it. Colony spreading was partially inhibited on 2.0% agar, and completely inhibited when the agar concentration exceeded 2.5%. We then examined the behavior of motile cells at the leading edge zones of colonies grown on 0.3%, 0.5%, and 1.5% agar media by light microscopy (Fig 1B). Because cells grown on 0.3% agar media moved independently in three dimensions within a thick fluid layer, we were not able to obtain well-focused images. The behavior looks like swimming motility. Cells on 0.5% agar media were observable in a single plane, and often formed small groups that moved together. These groups were unstable and frequently changed their members and shape during movement. On 1.5% agar media, most cells formed groups in which the cells were closely aligned along their long axis; these cells often formed bundles (Fig 1B). The number of cells in these clusters varied from a few to over thousands, and the larger clusters contained stacks of cells (Fig 1B; lower-right panel). Neither these densely packed clusters nor large clusters were observed at the leading edge zones of colonies grown on 0.3% and 0.5% agar media. Among these clusters, larger clusters moved forward smoothly, while single cells and small clusters moved very slowly or not at all (S1 Movie and S2 Movie). Thus, cluster formation appears to facilitate cell movement on the surface of 1.5% agar media. While the clusters were moving forward, some cells often fell away from the clusters, suggesting that these detached cells comprise static single cells and small clusters at the leading edge zone. Swarm bacteria often exhibit elongated cellular morphology during swarming motility [5, 6, and references therein]. However, *Paenibacillus* sp. did not show elongated cell morphology on 1.5% agar media.

**Fig 2:**
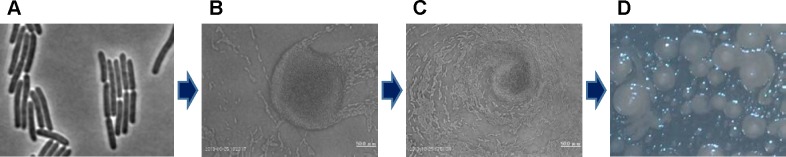
Progression of motility on 1.5% agar media. After inoculation, *Paenibacillus* sp. formed small clusters of cells (A), which grew up to visible tiny colonies. These colonies then moved around actively at speed 3.6 μm sec^-1^ (B). When the colony density became high, colonies began to rotate in a vortex like formation (C). The rotation speed of the vortices gradually decreased and finally static round colonies were formed (D). The photographs are not to scale.

We next investigated how the formation of moving cell clusters led to the colony scattering phenotype on 1.5% agar. For this purpose, a suspension of *Paenibacillus* sp. was spotted onto 1.5% agar and the process of colony formation analyzed under a stereo microscope (S3 Movie). During the first few hours post-inoculation, multiple tiny colonies appeared at the inoculation site, which then, while growing, moved around actively over the surface of the media. The speed of the moving colonies was 3.6 μm sec^-1^ (an average of 12 colonies was monitored for 10 min). Each moving colony appeared to have polarity; a fixed forward region led colony movement. The shape of moving colonies was not constant and sometime became very long. Moving colonies sometimes coalesced and divided. Some colonies then began to rotate and form vortices. Vortex formation began at the inoculation site, which contained a high cell density, and all colonies formed vortices as the colony density increased. Both clockwise and counterclockwise vortices were observed. The rotation speed of the vortices gradually decreased until static colonies were formed. These observations show that moving microscopic clusters of cells grow up to form visible colonies that move actively on hard agar (Fig 2). We called these moving colonies “wandering colonies”, as first described by Henrichsen [3]. Each wandering colony then rotated and finally became a static round colony (Fig 2). These unusual behaviors are the reason why *Paenibacillus* sp. forms many discrete colonies on hard agar media. Moving colonies have also been described for *Paenibacillus vortex*, which forms a highly branched colony pattern on hard agar media [24]. At the tips of growing branches, tens to thousands of cells form a spiraling colony that moves forward on hard agar media [24]. However, unlike for *Paenibacillus vortex*, *Paenibacillus* sp. colonies moved forward without spiraling as wandering colonies; the formation of spiraling colonies by *Paenibacillus* sp. was observed in transition from the active moving phase to the sessile phase (S3 Movie). Thus, colony movement and spiraling are separate entities in *Paenibacillus* sp. at least under the growth conditions tested herein.

### Draft genome sequencing of *Paenibacillus* sp. NAIST15-1

The entire genome of *Paenibacillus* sp. was sequenced using a massively parallel sequencing platform, MiSeq (Illumina). A total of 1.7 Gb of 300 base paired-end reads were assembled into 42 contigs (>1 kb) using CLC genomic workbench ver. 6.5 (CLC bio, Qiagen). The N_50_ value, which is a statistical measure of average length of contigs, was 264,241 bp and the longest contig was 824,207 bp. The draft genome of *Paenibacillus* sp. contains 6,768,284 bp, with a G+C content of 46.3%. The genome comprises 6,034 coding sequences, 5 ribosomal RNA partial sequences, and 78 transfer RNAs.

**Fig 3:**
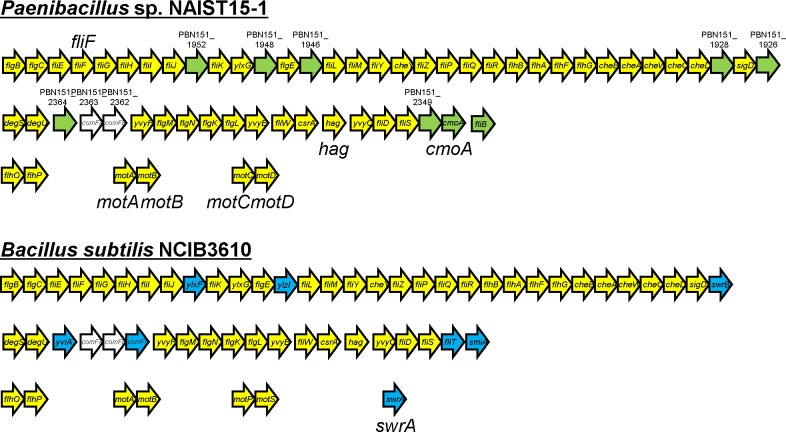
Organization of flagellar genes in *Paenibacillus* sp. and *Bacillus subtilis*. Five flagellar gene loci are shown for each bacterium. Flagellar structural and regulatory genes are shown in yellow. Genes specific for *Paenibacillus* sp. and *Bacillus subtilis* are shown in green and blue, respectively. Genes not directly linked to motility are in white. Genes described in text are denoted by large letters. The gene map is not to scale.

Flagellar genes were identified at five different loci within the *Paenibacillus* sp. genome. The largest cluster was the 30 kb *fla*/*che* operon, which contains genes for the flagellar hook-basal body complex, chemotaxis proteins, and an alternative sigma factor, σ^D^ (Fig 3). Another large flagellar gene cluster contains genes that encode anti-σ^D^ FlgM, the CsrA translational regulator, flagellin, and the filament cap. The other loci were the *flhOP* operon, which is probably required for hook assembly, and two stator operons (*motAB* and *motCD*). The composition and organization of these flagellar gene clusters were very similar to that of a Gram-positive model bacterium, *Bacillus subtilis* (Fig 3). These bacteria are expected to have a similar system for flagellar formation. However, *B*. *subtilis* has SwrA, which is required for transcriptional activation of the *fla*/*che* operon [30], but no *swrA* homolog gene was found in the *Paenibacillus* sp. genome. *swrA* is located between *ftsEX*-*ctpB* and *uvrBA* in the *B*. *subtilis* genome. Interestingly, these homologs are found upstream and downstream of *motAB* in the *Paenibacillus* sp. genome (S3 Fig).

Type IV pili are involved in motility in some bacteria [31]. Here, pili-related genes were also identified at two loci (S4A Fig). These gene products were similar to Flp pilus assembly proteins, Tad pilus assembly protein, and pre-pilin peptidase. However, our homology search did not identify all of the genes involved in Type IV pili biosynthesis, such as genes for pilin, and inner membrane core protein.

### Flagella are essential for motility on hard agar media

**Fig 4:**
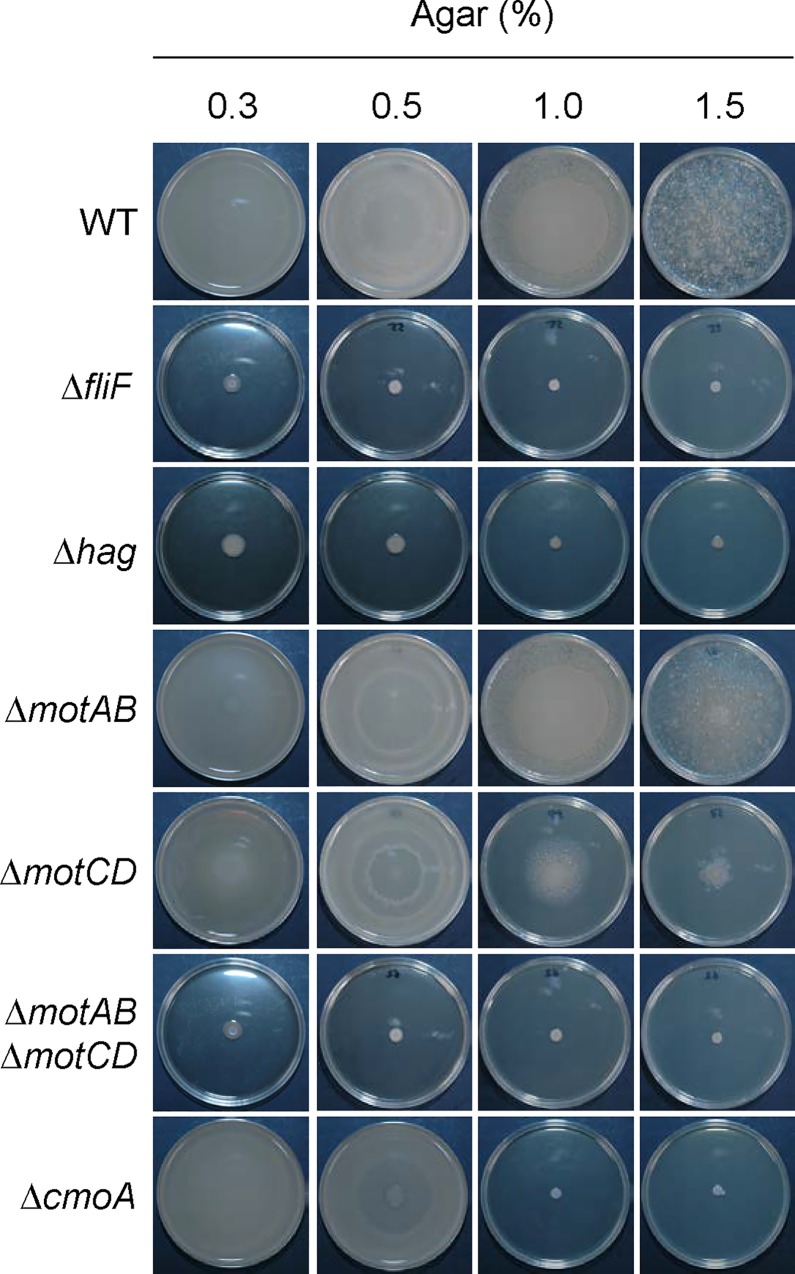
Motility-defective mutants. Wild-type and mutant strains were inoculated onto the center of 2×YT solidified with the indicated agar concentrations. Plates were incubated at 37°C for 18 h. Plate diameter, 9 cm.

Genome sequencing suggests that *Paenibacillus* sp. possesses at least two potential organelles for motility: flagella and pili. To determine which might be responsible for motility, we carried out gene disruption. Flagellar genes *fliF* and *hag* encode the M ring of the flagellar basal body and flagellin, respectively. We constructed bacteria harboring in-frame deletions of *fliF* or *hag* using the pMAD plasmid [32]. The growth rates of the *fliF* and *hag* mutants in 2×YT liquid media were indistinguishable from those of the wild-type strain (S5 Fig). The motility of these disruption mutants was then tested on media containing agar concentrations varying from 0.3% to 1.5%. Disrupting *fliF* and *hag* completely abolished motility on all agar media (Fig 4). The deleted *fliF* and *hag* alleles were then restored using a pMAD plasmid carrying the wild-type sequence of *fliF* or *hag*. The resulting complemented strains exhibited normal motility (comparable with that of the wild-type strain) (S6 Fig). On the other hand, deletion of putative pili genes (*PBN151_298*, *PBN151_299*, *PBN151_1478*, and *PBN151_1479*) did not affect motility on 2×YT plates (S4B Fig). These results clearly show that flagella are required for motility on both soft and hard agar media.

**Fig 5:**
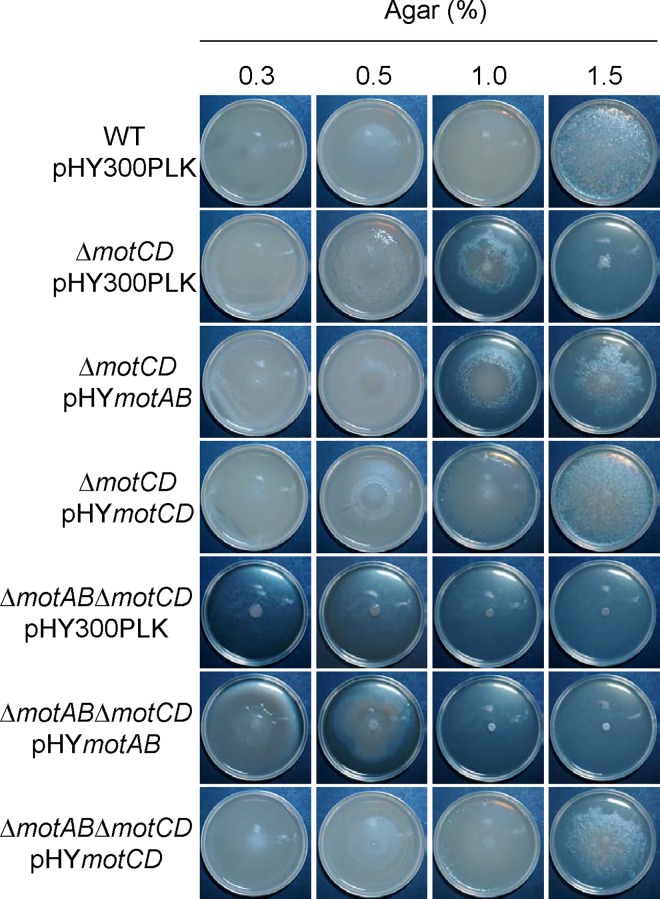
Complementation test of *motCD* and *motABmotCD* mutants. Multicopy plasmids carrying *motAB* (pHY*motAB*) or *motCD* (pHY*motCD*) were introduced into the *motCD* deletion mutant or the *motABmotCD* deletion mutant, and the motility ability of resultant strains was examined at 37°C for 18 h on 2×YT agar media. pHY300PLK is a parental plasmid. Plate diameter, 9 cm.

Flagellar rotation depends on the stator protein complex [33]. *Paenibacillus* sp. possesses two operons for stator proteins, *motAB* and *motCD*. MotA and MotC are 35.6% identical, whereas MotB and MotD are 30.6% identical (S7A Fig). To test whether flagellar rotation is required for motility, we constructed disruption mutants of these operons. Whereas disrupting *motAB* did not affect motility on all agar media tested, disrupting *motCD* prevented motility on 1.0% and 1.5% agar media (Fig 4). Disrupting both *motAB* and *motCD* completely abolished motility on all agar media tested (Fig 4). We then cloned *motAB* or *motCD* in the multicopy plasmid pHY300PLK [34] and carried out complementation tests. Introduction of multicopy *motAB* (pHY*motAB*) only partially restored motility to the *motCD* mutant, whereas introduction of pHY*motCD* fully restored motility to the *motCD* mutant (Fig 5). We also carried out complementation tests using the *motABmotCD* quadruple mutant. Introduction of pHY*motAB* restored motility to the *motABmotCD* mutant only on 0.3% and 0.5% agar media whereas introduction of pHY*motCD* restored motility to the *motABmotCD* mutant on all agar media tested (Fig 5). These results indicate that either *motAB* or *motCD* is sufficient for motility on 0.3% and 0.5% agar media, but that only *motCD* can facilitate motility on 1.0% and 1.5% agar media. The requirement for both flagella and stator proteins indicates that flagellar rotation generates the impetus for motility on both soft and hard agar media.

**Fig 6:**
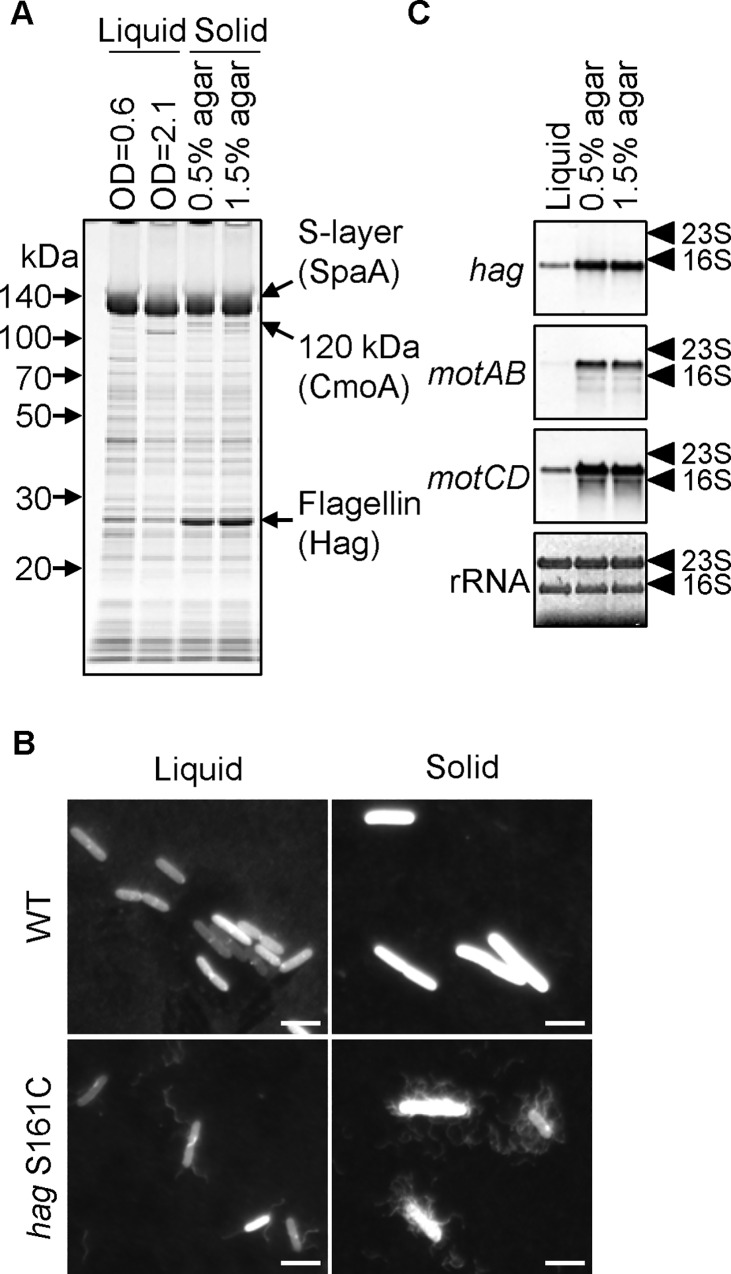
Flagellar formation is induced by surface growth on solid media. (A) Flagellin levels. The wild-type strain was grown to OD_600_ = 0.6 or 2.1 at 37°C in 2×YT liquid media or at 37°C for 5 h on 2×YT/0.5% or 2×YT/1.5% agar media. Cells were collected from each culture and resuspended in 10 mM Tris buffer. The number of cells in each sample was adjusted according to the OD_600_ value. Cell-surface associated proteins were extracted by boiling in 0.1% SDS. The extracted proteins were then separated by SDS-PAGE, and strained with CBB. S-layer, CmoA, and flagellin proteins are indicated by arrows. The positions of molecular weight markers are shown. (B) Fluorescent staining of flagella. Wild-type and *hag* S161C (P148) strains were grown to OD_600_ = 0.6 in 2×YT liquid media or grown for 5 h on 2×YT/1.5% agar plates at 37°C. Cells were collected and incubated with Alexa Fluor 594 C5 maleimide in the dark. After a brief wash, cells were observed under a fluorescence microscope. Scale bar, 5 μm. (C) Comparison of flagellar gene expression. Total RNA samples were isolated from wild-type cells grown in liquid media, on 0.5% agar, or on 1.5% agar media. Transcription of the indicated genes was analyzed by Northern blotting. The positions of 23S and 16S ribosomal RNAs (rRNAs) are indicated by arrow heads. rRNAs stained with methylene blue are shown as a loading control.

The number of flagella is a key factor for robust motility [35–38]. Increasing the number of flagella allows bacteria to swarm on harder agar media or to swim through more viscous media. We hypothesized that increasing the number of flagella might be required for motility on hard agar media. To test this hypothesis, we first carried out fractionation experiments to identify the flagellin protein in *Paenibacillus* sp. protein extracts. Approximately 8 × 10^6^ cells were spread over the surface of a 9 cm diameter 1.5% agar plate. After a 5 h incubation at 37°C, *Paenibacillus* sp. formed many moving colonies on 1.5% agar plates. These bacterial cells were suspended in buffer, collected, and the bacterial proteins separated into three fractions: secreted proteins, cell-surface associated proteins, and cellular proteins, as described in Methods. These fractions were then analyzed by SDS-PAGE (S8 Fig). We expected that flagellin would mainly be restricted to the cell-surface associated protein fraction, in which two strong protein bands (150 kDa and 28 kDa) were detected on SDS-PAGE gels (S8 Fig). LC-MS/MS analysis identified these two proteins as the S-layer protein (SpaA; Mw 111.5 kDa) and flagellin (Hag; Mw 29.6 kDa) (S9 Fig). The size of the S-layer protein on SDS-PAGE was much greater than its deduced molecular weight. The S-layer protein of *Paenibacillus alvei* is glycosylated [39, 40]. Also, *Paenibacillus* sp. possesses a putative S-layer glycosylation gene cluster (*PBN151_2313* to *PBN151_2300*] in its genome. These observations suggest that the S-layer protein in *Paenibacillus* sp. is also glycosylated. The size of the flagellin protein on SDS-PAGE was very similar to its deduced molecular weight. These results confirm that flagellin is a major cell-surface associated protein. We then compared flagellin levels in the cell-surface associated protein fraction under three different growth conditions: liquid media and solid media containing 0.5% or 1.5% agar. Since 0.3% agar media are fragile, we used liquid media rather than 0.3% agar media for this analysis. Flagellin levels in 0.5% agar and 1.5% agar were the same (Fig 6A); however, those in liquid media were much lower at both the exponential- and early stationary growth phases (Fig 6A). To confirm this result, we tried to visualize flagellar filaments on cells grown in liquid or 1.5% agar media. To achieve this, a TCA (Ser) to TGC (Cys) substitution was introduced into the 161st codon of *hag* on the genome, which would allow the flagellin filaments to be labeled with a sulfhydryl-reactive fluorescent dye. The S161C substitution did not interfere with flagellin function because the *hag* S161C strain showed normal motility (S6 Fig). After fluorescent labeling, filaments were visible in the *hag* S161C strain but not in the wild-type strain (Fig 6B), indicating that the visualized filaments were indeed flagella. Consistent with the results of SDS-PAGE analysis, cells grown in liquid media had a few flagella, whereas those grown on 1.5% agar media exhibited peritrichous hyperflagellation (Fig 6B). We further compared the transcription of flagellar genes (*hag*, *motAB*, and *motCD*) in liquid, 0.5% agar, and 1.5% agar cultures. Northern blot analysis revealed that transcription of *hag*, *motAB*, and *motCD* was low in liquid, and strongly induced in 0.5% and 1.5% agar media (Fig 6C). Thus, hyperflagellation was supported by the transcriptional induction of flagellar genes on solid media. These results demonstrate that flagellar formation is strongly induced in response to growth on the surface of media containing agar at concentrations of 0.5% or above. Wandering colony formation was observable on media containing >1.0% agar, as described above. Flagellin levels and flagellar gene expression between 0.5% and 1.5% agar media were the same, indicating that flagellin levels *per se* are not a discriminator of wandering colony formation. Additional factors may be required for wandering colony formation on hard agar media.

### Identification of *cmoA*

**Fig 7:**
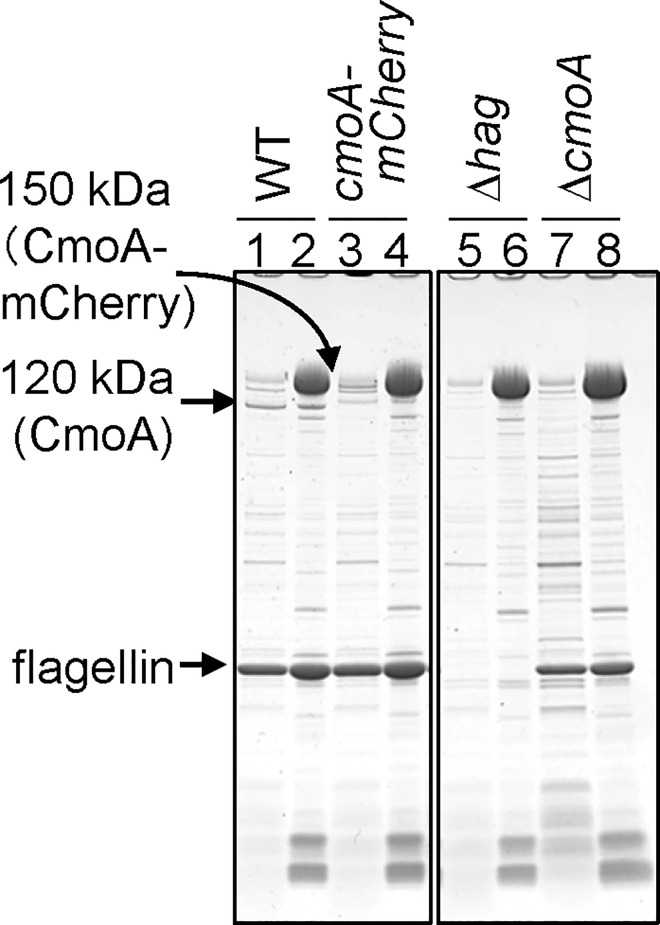
CmoA levels in the wild-type and mutant strains. Strains (WT, wild-type; *cmoA-mCherry*, P205, Δ*hag*, P261; Δ*cmoA*, P198 in Table 1) were grown at 37°C for 5 h on 1.5% agar media. The secreted protein (lanes 1, 3, 5, and 7) and cell-surface associated protein (lanes 2, 4, 6, and 8) fractions were prepared and separated by SDS-PAGE. The positions of CmoA-mCherry, CmoA, and flagellin are indicated by arrows.

When cellular proteins were fractionated, flagellin was observed not only in the cell-surface associated protein fraction but also in the secreted protein fraction in the wild-type strain (Fig 7, lanes 1 and 2). We found that one large, unknown protein (120 kDa) exhibited the same distribution as flagellin (Fig 7, lanes 1 and 2). Moreover, the 120 kDa protein was induced on solid media, as observed for flagellin (Fig 6A). Interestingly, the level of the 120 kDa protein was greatly reduced in the *hag* mutant (Fig 7, lanes 5 and 6). Therefore, we hypothesized that the 120 kDa protein might be involved in motility on hard agar media.

LC-MS/MS analysis revealed that the 120 kDa protein was the product of *PBN151_2348* (S9 Fig), which is located downstream of the flagella gene cluster (Fig 3). *PBN151_2348* is hereafter called *cmoA* (see below). To confirm that the 120 kDa protein was the product of *cmoA*, we constructed two new strains: an in-frame deletion mutant of *cmoA* and a *cmoA-mCherry* fusion strain. The fractionation analysis of cellular proteins revealed that deleting *cmoA* caused the 120 kDa protein to disappear from both the secreted and cell-surface associated protein fractions (Fig 7, lanes 7 and 8). The introduction of the *mCherry* tag into the 3-terminus of *cmoA* resulted in the disappearance of the 120 kDa protein and the appearance of a new 150 kDa protein (Fig 7, lanes 3 and 4). Unfortunately, the 150 kDa protein was hidden behind the abundant S-layer protein in the cell-surface associated protein fraction, and was only observable in the secreted protein fraction. The size of this protein (150 kDa) was consistent with the deduced molecular weight of the CmoA-mCherry fusion protein. These results confirm that the 120 kDa protein is the product of *cmoA*.

The motility assay revealed that the *cmoA* deletion mutant spread on 0.3% and 0.5% agar media but not on 1.0% and 1.5% agar media (Fig 4). The complemented strain exhibited normal motility, comparable with that of the wild-type strain (S6 Fig). Thus, CmoA is specifically required for motility on hard agar media. SDS-PAGE analysis showed that the *cmoA* mutant grown on 1.5% agar media expressed high levels of flagellin (Fig 7, lanes 7 and 8). Indeed, when water was poured on *cmoA* mutant cells grown on 1.5% agar, the cells immediately moved actively in water (S4 Movie). These results indicate that the *cmoA* mutation causes defects in motility on hard agar media without preventing flagellar formation. Based on these observations, we designated this gene *cmoA* (colony movement gene A).

**Fig 8:**
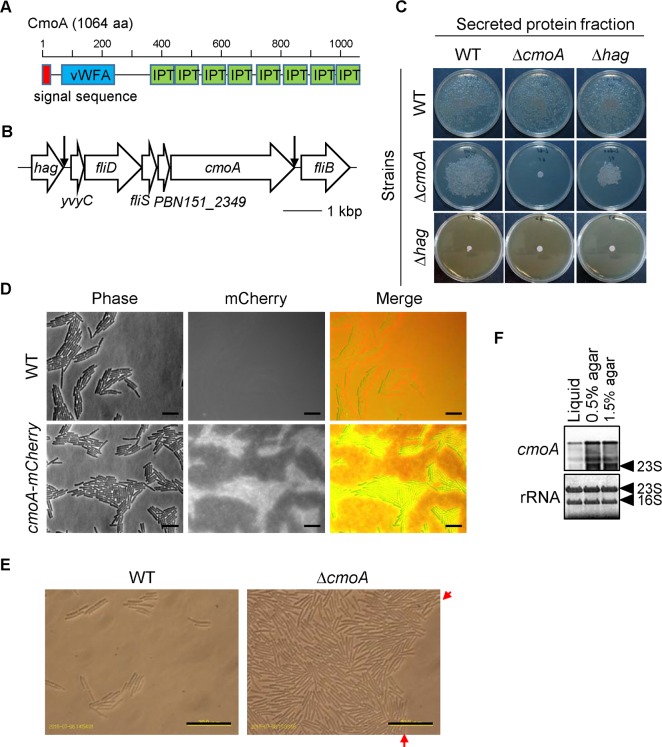
CmoA has extracellular functions. (A) Schematic drawing of the structure of CmoA. The positions of the N-terminal signal sequence (required for secretion), the vWFA domain, and the IPT/TIG domains (IPT) are shown. (B) Gene organization of the *cmoA* locus. The positions of the transcriptional terminator sequences are indicated by downward arrows. (C) Extracellular complementation of the *cmoA* mutant. Secreted protein fractions were prepared from plate cultures of the indicated strains and spread over the surface of 1.5% agar plates. Wild-type and mutant strains were then inoculated onto the center of those plates and grown at 37°C for 18 h. Strains: WT, wild-type; Δ*cmoA*, P260; and Δ*hag*, P261 in Table 1. Plate diameter, 9 cm. (D) Localization of CmoA. Wild-type and *cmoA-mCherry* (P205) strains were spotted onto 1.5% agar media and grew until small colonies moved out from the inoculation site. Coverslips were placed directly onto the moving colonies on the plates and examined under a fluorescence microscope. Merged phase-contrast (false-colored red) and mCherry (false-colored green) images are shown. Scale bar, 5 μm. (E) The leading edge zones of expanding wild-type and *cmoA* mutant colonies on 0.6% agar media. The swarm colonies were spreading from the upper left to the lower right in these photographs. The leading edge of the *cmoA* mutant swarm colony is indicated by arrows. Scale bar, 20 μm. F. Northern blot analysis of *cmoA*. Total RNA samples were isolated from wild-type cells grown in liquid media, on 0.5% agar media, or on 1.5% agar media. The positions of 23S and 16S rRNAs are indicated by arrow heads. rRNAs stained with methylene blue are shown as a loading control.

CmoA is a large protein comprising 1,064 amino acids and contains an N-terminal signal sequence required for secretion, a single vWFA (von Willebrand factor type A) domain, and eight tandem IPT/TIG (immunoglobulin, plexins, transcription factors-like/transcription factor immunoglobulin) domains (Fig 8A). The vWFA domain is observed in cell adhesion and extracellular matrix proteins in eukaryotes [41], while the IPT/TIG domain is observed in cell surface receptors and transcription factors in eukaryotes [42]. However, their function in bacteria is unknown. By protein blast search on the National Center for Biotechnology Information (http://blast.ncbi.nlm.nih.gov), we identified 29 CmoA homologs; 24 were found in *Paenibacillus* spp., and the others were found in *Brevibacillus thermoruber*, *Aeribacillus pallidus*, *Domibacillus indicus*, *Cohnella thermotolerans*, and *Clostridium* sp. BL8. Except for *Clostridium* sp. BL8, these are *Paenibacillus* and its closest relatives. Among them, four species are reported to exhibit wandering colonies or the colony scattering phenotype in literatures: *P*. *vortex* [24], *P*. *alvei* CCM2051T [43], *P*. *assamensis* [44], and *Paenibacillus* sp. Y412MC10 [45], though while the colony phenotype of the other species is unknown. On the other hand, *P*. *dendritiformis*, *P*. *polymyxa*, *P*. *larvae*, all of which do not form wandering colonies, have no CmoA homolog. In *Paenibacillus* sp. *cmoA* is expected to constitute an operon with upstream genes *yvyC*, *fliD*, *fliS*, and *PBN151_2349* in the flagellar gene cluster because there is no terminator sequence between these genes (Fig 8B). Among them, FliD and FliS are similar to the filament cap protein and the flagellar type III export chaperon specific for flagellin, respectively. YvyC and PBN151_2349 are unknown small proteins (111 and 99 amino acids, respectively). Except for *PBN151_2349*, the *yvyC-fliD-fliS-(PBN151_2349)-cmoA* operon is conserved in *Paenibacillus vortex*, *Paenibacillus alvei* CCM2051T, *Paenibacillus* sp. Y412MC10, and *Paenibacillus assamensis* that form wandering colonies. Each bacterium has a different small gene at the position corresponding to *PBN151_2349*. *yvyC*, *fliD*, *and fliS* are also conserved as members of the flagellar gene operon, *yvyC*-*fliD*-*fliS-fliT*, in *Bacillus subtilis* [46]. These observations indicate that *cmoA* may be a member of flagellar regulon.

### The role of CmoA in motility

CmoA has the N-terminal signal sequence required for secretion, and was detected in extracellular protein fractions, indicating that CmoA might have an extracellular function. To address this, we tested whether the *cmoA* mutant exhibited extracellular complementation. The secreted protein fraction was prepared from the wild-type strain grown on 1.5% agar, sterilized by passing through a membrane filter, and spread over the surface of a 1.5% agar plate. When the *cmoA* mutant was inoculated onto the center of the plate, it spread over almost all the plate within 18 h of incubation (Fig 8C). In contrast, the *comA* mutant did not spread on 1.5% agar plates supplemented with the secreted protein fraction prepared from the *cmoA* mutant (Fig 8C). As described above, the *hag* mutant exhibited decreased CmoA (Fig 7). The secreted protein fractions derived from the *hag* mutant restored motility to the *cmoA* mutant only partially (Fig 8C). Those secreted protein fractions did not affect the motility of the wild-type strain, and did not restore motility to the *hag* mutant (Fig 8C). These results indicate that extraneous CmoA can restore motility to the *cmoA* mutant. Thus, CmoA plays an extracellular function in terms of motility on hard agar media.

We next examined the localization of CmoA using the *cmoA-mCherry* strain. The CmoA-mCherry fusion is functional because the *cmoA-mCherry* strain exhibits normal motility (S6 Fig). To examine its localization during motility on hard agar media, the *cmoA-mCherry* strain was spotted onto 1.5% agar plates and grown until small colonies moved out from the inoculation site. Coverslips were placed directly onto the moving colonies and examined under a fluorescence microscope (Fig 8D). The bacterial cells showed strong fluorescence emitted by mCherry, which appeared between cells and enveloped clusters of cells. Weak fluorescence was also observed across the surface of the media, indicating that a small part of CmoA diffuses into the media during colony movement. No fluorescence signal was observed in wild-type strain under the same conditions.

We tried to examine the effect of the *cmoA* mutation on cellular behavior. However, we were unable to examine cellular behavior on 1.5% agar media. This is because motility mutants of *cmoA* and *hag* did not move when spread over the surface of 1.5% agar media; rather they formed cell aggregates by dividing within the inoculated spots. The shape of the aggregates was quite similar to that of moving cell clusters formed by the wild-type strain (S10 Fig). The *cmoA* mutant was able to spread on media containing up to 0.6% agar. The clusters formed by the wild-type strain on 0.6% agar media tended to be larger than those formed on 0.5% agar (S11 Fig). Therefore, we compared cellular behavior of both the wild-type strain and the *cmoA* mutant on 0.6% agar media using video light microscopy. The density of the wild-type cells at the leading edge zone of expanding colonies was very low (Fig 8E), and the cells frequently formed small clusters of several cells (S5 Movie). The clusters of wild-type cells moved more rapidly and smoothly than single cells, and moved forward into the unexplored region of agar media without stalling (S5 Movie). By contrast, the *cmoA* mutant formed a clear leading edge with a high cell density (Fig 8E). *cmoA* mutant cells in the area immediately inside the edge moved but did not form cell clusters (S6 Movie). When *cmoA* mutant cells reached the leading edge, some stalled and others turned back. The behavior of the *cmoA* mutant is very similar to that of other swarm bacteria, in that the cells are active within the swarming colonies but stall at the swarming edge on hard agar media. Swarming bacteria attract water from agar media and swarmer cells within the swarm colony move in the resulting fluid layer [7–10]. However, the fluid layer at the edge is very thin and cannot support cell movement until sufficient fluid is supplied from inside the swarm colony [10, 47, 48]. Thus, our observations indicate that expansion of the *cmoA* mutant probably depends on the fluid layer, as observed for other swarm bacteria. The *cmoA* mutant is probably unable to move into the unexplored region until sufficient fluid is supplied from inside the swarm colony. The leading edge region of the wild-type strain had very low cell density. Since a high cell density is thought to be important for extracting water from agar media and maintaining the resulting fluid [10], wild-type cells do not appear to acquire water in the same manner, as the *cmoA* mutant and other swarm bacteria. In fact, single cells of the wild-type strain were able to move slowly by themselves at the leading edge zone on 0.6% agar media (S5 Movie). These observations indicate that the wild-type strain has a water acquisition mechanism that does not depend on cell population, and that the *cmoA* mutant loses this mechanism. One possible explanation is that cell associated CmoA may facilitate extraction of water from the agar media and/or smooth the cell surface interface, which enables the wild-type strain to move on harder agar media.

We hypothesized that exogenous CmoA may stimulate motility of other bacteria. To test this possibility, we examined motility of *B*. *subtilis*, *Paenibacillus alvei*, and *E*. *coli* on plates supplemented with or without CmoA. The result showed that exogenous CmoA slightly enhanced *B*. *subtilis* motility: these cells formed slightly larger swarm colonies on 1% agar plates supplemented with the *Paenibacillus* sp. wild-type lysate than on plates supplemented with the *cmoA* mutant lysate (S12 Fig). A similar tendency was observed for the *B*. *subtilis srfAC* mutant, which does not produce the surface active compound surfactin involved in swarming motility, on 0.8% agar [49]. However, these effects were not very marked: therefore, we cannot conclude that CmoA stimulates *B*. *subtilis* motility. Exogenous CmoA did not affect motility of *Paenibacillus alvei* and *E*. *coli* (S12 Fig).

### Low wetness is required for wandering colonies

**Fig 9:**
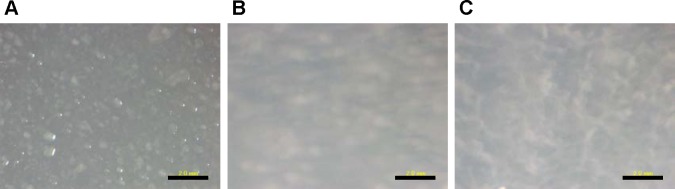
Wandering colonies are sensitive to wetness. Dilution of wild-type culture was spread over the surface of a 1.5% agar plate and incubated at 37°C until wandering colonies appeared (A). Water was gently poured onto the plate (B), and then the plate was gently shaken three times (C). Scale bar, 2 mm.

We compared *cmoA* expression in liquid and agar cultures. Northern blot analysis revealed that *cmoA* transcription was low in liquid culture and strongly induced in 0.5% and 1.5% agar cultures (Fig 8F), which was the same with flagellar genes, *hag*, *motAB* and *motCD* (Fig 6C). The size of *cmoA* transcript was much bigger than 23S rRNA (Fig 8E), suggesting that *cmoA* is probably cotranscribed with upstream genes *yvyC*, *fliD*, and *fliS* as expected from DNA sequence (Fig 8B). Wandering colonies were observable on media containing >1.0% agar. However, expression of *cmoA* and flagellar genes was the same between 0.5% and 1.5% agar media, indicating that the induction of *cmoA* and flagellar genes is insufficient to support wandering colony formation. As shown in S5 and S6 Movies, wild-type cells formed moving cell cluster in a CmoA-dependent manner on 0.6% agar. However, these moving clusters did not grow to visible wandering colonies on 0.6% agar media, suggesting that moving clusters are less stable on 0.6% agar than on 1.5% agar. We hypothesized that wetness might affect the stability of wandering colonies. To test this hypothesis, a small amount of water was gently poured on wandering colonies grown on 1.5% agar and cellular behavior was immediately observed. We found that wandering colonies disassembled easily and quickly in water (Fig 9), and individual cells moved randomly in water (S7 Movie). These observations indicate that wandering colonies are very sensitive to wetness and cannot maintain their shape in high wet conditions. These observations indicate that low wet conditions are required not only to induce *cmoA* and flagellar gene expression but also to maintain wandering colonies. Thus, *Paenibacillus* sp. forms wandering colonies specially to move on low wet surfaces.

## Discussion

Here, we describe cooperative motility behavior of *Paenibacillus* sp. NAIST 15–1 and its genetic analysis. *Paenibacillus* bacteria have commonly been isolated from diverse environmental samples, such as soil, hot springs, blood samples, honeybees, and plants [14–18, 22, 44, 45]. However, their genetic studies have been limited due to the difficulty in their genetic manipulation. We demonstrated that *Paenibacillus* sp. was amenable to genetic manipulation. Thus, this strain will be useful for genetic analysis of *Paenibacillus* bacteria. *Paenibacillus* sp. exhibited distinct types of flagella-driven motility, which was dependent on the agar concentration. Individual cells swam in liquid media or in a thick fluid layer on 0.3% agar media, but exhibited hyperflagellation and formed small moving groups, when grown on the surface of 0.5% agar media. Bacterial motility is often inhibited in solid media containing agar at concentrations above 1%. However, *Paenibacillus* sp. formed wandering colonies, which moved across the surface of media containing >1.5% agar.

We showed that flagella are essential for the motility of *Paenibacillus* sp. Despite its special motility behavior, the organization and composition of flagellar genes in *Paenibacillus* sp. are quite similar to those in *B*. *subtilis*. Flagella formation by *Paenibacillus* sp. was induced on hard agar media, which led to peritrichous hyperflagellation. Hyperflagellation was supported by the transcriptional induction of flagellar gene expression. Transcriptional induction of flagellar gene expression in response to growth on hard agar media is also observed in other swarm bacteria including *B*. *subtilis*, *Vibrio* spp., *Proteus mirabilis*, and *Serratia marcescens* [36, 50–53]. An artificial increase in the number of flagella allows bacteria to swarm on harder agars or to swim through more viscous media in *B*. *subtilis*, *Salmonera enterica*, *Proteus mirabilis*, *Pseudomonas aeruginosa* [35–38]. Thus, an increase in the number of flagellar is a key factor for motility on hard agar media. Flagellar stators generate the torque required for flagellar rotation using an electrochemical gradient of H^+^ or Na^+^ across the cytoplasmic membrane [33]. *Paenibacillus* sp. possesses two sets of flagellar stators, MotAB and MotCD. The gene disruption experiment showed that both MotAB and MotCD facilitate motility on 0.3% and 0.5% agar, but only MotCD facilitates motility on 1.0% and 1.5% agar. Multicopy MotAB was unable to restore motility to the *motCD* mutant on hard agar media. These observations indicate that MotCD plays a specific role in motility on hard agar media at least under the conditions tested herein. Some bacteria harbor multiple stators that play distinct roles in motility. *Vibrio* spp. has two types of flagella: polar and lateral. Polar flagella are driven by the Na^+^-dependent stator and provide the impetus for swimming motility [54]. Lateral flagella are driven by the H^+^-dependent stator and provide the impetus for swarming motility [54]. The formation of lateral flagella and their stators is transcriptionally induced in response to growth on hard agar media [53]. However, unlike *Vibrio* spp., transcription of both *motAB* and *motCD* operons of *Paenibacillus* sp. was induced on hard agar. A recent study shows that *Paenibacillus* sp. TCA20 also has two sets of stator operons, *motA1motB1* and *motA2motB2* [55]. Phylogenetic analysis of MotB1 and MotB2 along with other MotB stator subunits showed that MotB2 is highly similar to H^+^-dependent MotB stators, whereas MotB1 belongs to a different stator cluster from known H^+^-dependent or Na^+^-dependent MotB stators [55]. The MotB1-type stators are unique to *Paenibacillus* bacteria, and the MotA1MotB1 stator is suggested to use Ca^2+^ or Mg^2+^ as coupling ions [55]. Phylogenetic analysis showed that MotB of *Paenibacillus* sp. was classified into the group of MotB1-type stators, whereas MotD was classified into the group of H^+^-dependent MotB stators (S7B Fig). These observations indicate that MotAB and MotCD may use different coupling ions for flagellar rotation. Although at present the reason for the functional difference between MotAB and MotCD is unclear, the finding that flagella and stator proteins are required for motility strongly suggests that flagella rotation provides the impetus for *Paenibacillus* sp. motility on both soft and hard agar media.

Swarming motility is defined as flagella-driven group motility on a solid surface [3–6]. According to this definition, the motility by *Paenibacilus* sp. on hard agar media is classified as swarming motility, although it has unique characteristics. Since flagella rotation pushes a cell forward against the surrounding water, surface water is a critical element for swarming motility. Swarming bacteria on hard agar media can attract water to the surface from the agar matrix [7–10]. Cell growth (a high cell density), cellular secretions, and flagella rotation are thought to attract water to the surface, from where it diffuses throughout the swarming colony [7–11, 47, 48]. As a result, the swarming colony maintains a fluid layer within itself, in which each cell moves actively. However, the edge of the colony contains only a very thin layer of fluid [10]; thus swarmer cells stall there until fluid is supplied from the inside of the swarm colony [11, 47]. The flagella of stalled cells at the edge of the swarm colony frequently orient toward the unexplored region of the media and rotate; this likely pumps fluid outwards, thereby aiding expansion of the swarming colony [11, 47]. Thus, swarmer cells cannot move into unexplored regions of hard agar media without first expanding the fluid layer, and swarm bacteria usually formed single swarm colony when inoculated onto a single site on hard agar media. *Paenibacillus* sp. formed many clusters on hard agar media, which grew up to form visible wandering colonies. Cells in wandering colonies were associated with neighbor cells and did not move within wandering colonies. Cell clusters and wandering colonies were able to leave the fluid layer of the mother swarm colony and move around the unexplored region on hard agar media by themselves. As a result, *Paenibacillus* sp. formed multiple colonies even when inoculated into single site on hard agar media.

We identified CmoA as a protein specifically required for motility on hard agar media. CmoA is a large protein of 1,064 amino acids and comprises a single vWFA domain and eight tandem IPT/TIG domains. The vWFA and IPT/TIG domains are mainly conserved in eukaryotes and many of these domains are found in extracellular proteins such as cell adhesion proteins, extracellular matrix proteins, and cell surface receptors [41, 42]. Proteins containing these domains are also found in bacteria, but are not well characterized. The N-terminus of CmoA is a typical signal sequence for secretion. Indeed, CmoA was detected in extracellular fractions. Moreover, the motility of the *cmoA* mutant was restored in media supplemented with the secreted protein fraction derived from the wild-type strain, but not that from a *cmoA* mutant. Live imaging of cells expressing the CmoA-mCherry fusion protein plated on hard agar media showed that CmoA was mainly localized around the cells. CmoA-mCherry was present between the cells within clusters, but also covered entire clusters. These observations show that CmoA has an extracellular function, as observed for vWFA and IPT/TIG domain proteins in eukaryotes. The *cmoA* mutant was able to induce flagella in response to surface growth condition and moved on solid media containing up to 0.6% agar. However, its cellular behavior was quite different from that of wild-type cells. On 0.6% agar media, the wild-type strain formed small cell clusters that moved smoothly into the unexplored region of the media, whereas the *cmoA* mutant neither formed clusters nor moved smoothly into the unexplored region. Cells of the *cmoA* mutant moved actively inside the swarming colony, but stalled at the swarm edge and formed a clear leading edge. Interestingly, this behavior is similar to that observed for swarm cells of other bacteria such as *B*. *subtilis* and *E*. *coli* [11, 47–49], whose spread on hard agar media is dependent on the acquisition of fluid. Thus, the *cmoA* mutant seems to spread on hard agar media in a similar manner to other swarm bacteria. We also observed that the leading edge region of the wild-type strain had very low cell density. Under the condition, cell population-dependent water extraction from agar media is unexpected. In other word, *Paenibacillus* sp. has a water acquisition mechanism that does not depend on cell population. We propose that cell-surface associated CmoA may be involved in drawing water out of agar and/or smoothing cell surface interactions. Lacking this function, *cmoA* mutant cells may be unable to leave the mother swarming colony and move into the unexplored region on hard agar media. *Proteus mirabilis* secretes high molecular weight polysaccharide, which is responsible for extracting water from agar media [56]. *E*. *coli* secretes some osmolytes, probably lipopolysaccharides, to extract water [57]. Several swarm bacteria secrete surface active molecules, e.g. surfactin secreted by *B*. *subtilis* [49] and rhamnolipids by *Pseudomonas* spp. [58]. CmoA may be the first identified protein responsible for extracting water from agar media or for reducing surface tension to facilitate swarming motility.

We also observed that clusters of the wild-type cells moved much faster than single cells. Many swarm bacteria form cell clusters, called rafts, which also move faster than single cells in the fluid layer of swarm colonies. For instance, *B*. *subtilis* cells recruited to a raft moved with the group, whereas cells lost from a raft stop moving [49]. The formation of rafts is thought to facilitate movement on hard agar media partly by reducing viscosity/drag on individuals [12]. This though probably applies to cell clusters of *Paenibacillus* sp. As rafts of swarm bacteria are unstable and dynamically labile in terms of both members and shape, no specific substances or matrices appear to maintain rafts stability [5, 6, and references therein]. However, cell clusters of *Paenibacillus* sp. may have different features. Cell clusters of *Paenibacillus* sp. were able to leave the fluid layer of the mother colony and move alone into unexplored region. Moreover, on hard agar media containing >1% agar, those clusters grew up to form visible wandering colonies, in which cells are associated with neighbor cells. Therefore, the cell clusters of *Paenibacilus* sp. on hard agar media appear to be more stable than rafts of other swarm bacteria. We speculate that *Paenibacillus* sp. may have a mechanism for stabilizing cell clusters. Many swarm bacteria such as *B*. *subtilis* and *E*. *coli*, cannot move on media containing 1% agar. However, some bacteria, called robust swarmers, can move on media containing >1.5% agar [6, and references therein]. One such robust swarmer, *Proteus mirabilis*, forms highly organized multicellular rafts during swarming motility. Electron microscopy shows that adjacent cells in those rafts are connected via intercellular bundling of flagella [59]. Likewise, cells in wandering colonies of *Paenibacillus vortex* are also connected by filament networks, which are probably flagella. [24]. Since these electron microscope images were taken using fixation procedures, further confirmation using live cells will be required. On the other hand, these observations suggest that there may be a mechanism that stabilizes cell clusters in robust swarmers in which flagella may play an important role. The *cmoA* mutant did not form cell clusters and did not move on hard agar media containing >1% agar. CmoA was located around cells and enveloped cell clusters. Based on these observations, we speculate that CmoA may also play a role in stabilizing cell clusters. For example, cell-surface associated CmoA may interact with flagella, which form cell-cell connections. The social Amoeba, *Dictyostelium discoideum* forms multicellular fruiting bodies in response to nutrient exhaustion. During this process, cell-cell adhesion is mediated by binding between extracellular proteins TgrB1 and TgrC1 via mutual IPT/TIG domains [60]. CmoA itself may form cell-cell connection via its multiple IPT/TIG domains. However, cell-cell connections that support wandering colony formation are completely different from stable cell-cell connections observed in bacterial biofilms [61]. Wandering colonies were easily disassembled when water was added. The cell-cell connection in wandering colonies may be fragile or sensitive to wetting. Further work will be required to determine how *Paenibacillus* sp. forms wandering colonies and to examine role of CmoA in wandering colony formation.

Professor Ben-Jacob’s group isolated and extensively analyzed pattern forming *Paenibacillus* bacteria [23–25, and references therein]. However, these strains were isolated as contaminants from a *B*. *subtilis* stock and their natural habitat is unclear. Here, we isolated *Paenibacillus* sp. from the rhizosphere of natural weeds. We found that this type of *Paenibacillus* bacteria was easily isolated from natural weed roots and associated soils (S13 Fig). The natural habitat of pattern-forming *Paenibacillus* bacteria seems to be soil and its related environment including the rhizosphere. This seems to be the reason why *Paenibacillus* sp. exhibits complex motility behavior. Soil includes various substances, and its surface properties vary. The water content in soil is often variable and is sometimes very low. Thus, soil bacteria appear to employ multiple motility systems to overcome these environmental challenges. For instance, a soil bacterium, *B*. *subtilis*, exhibits flagellar-dependent swimming and swarming motility [49]. Recently, two groups reported that *B*. *subtilis* also exhibits sliding motility under conditions in which flagella do not work [62, 63]. *Myxococus xanthus*, which does not have flagella, also exhibits two types of motility, individual gliding motility and social twitching motility, which have different advantages on different surfaces [64]. *Pseudomonas aeruginosa* exhibits flagella-dependent motility, Type IV pili-dependent twitching motility, and sliding motility [65]. The results presented herein reveal that *Paenibacillus* sp. has evolved to use flagella under a wider range of conditions. To overcome conditions of low wetness, *Paenibacillus* sp. forms multicellular wandering colonies. When wandering colonies encounter wet conditions, they quickly disassembled and individual cells can swim or swarm in the water layer. These properties will be advantageous for *Paenibacillus* sp., enabling it to disperse its cells rapidly and widely in soil.

## Methods

### Isolation of *Paenibacillus* sp. NAIST15-1

Plant weeds were uprooted from the ground within Nara Institute of Science & Technology (NAIST) and most of the soil around the roots removed. The plant roots were then cut, placed in 15 ml tubes, and soaked in 2 ml LB medium. After a brief vortex, the extract was incubated at 85°C for 10 min and then plated onto LB at appropriate dilutions. Twelve strains, all of which showed a colony scattering phenotype and antagonistic activity against *Fusarium oxysporum*, were isolated. Among these, *Paenibacillus* sp. NAIST15-1 was selected for analysis based on its strong growth and transformability. The 16S rDNA region was amplified from its genome DNA with primers 16S-F1 (5’-TTAGCGGCGGACGGGTGAGT) and 16S-R1 (5’-TGACGGGCGGTGTGTACAAG). Nucleotide data for partial 16S rDNA sequence has been submitted to the DDBJ/EMBL/GenBank databases under the accession number, LC185074.

### Strains and growth conditions

The *Paenibacillus sp*. strains used in this study are listed in Table 1. All strains were derivatives of *Paenibacillus* sp. NAIST 15–1. *Paenibacillus* sp. was grown in LB (LB Lennox, Difco) or 2×YT (16g l^-1^ tryptone (Difco), 10g l^-1^ yeast extract (Difco), 5g l^-1^ NaCl) medium. For plates, media were solidified with the indicated concentrations of agar (for Microorganism Culture, Nakalai Tesque). Single colonies were isolated on 2.5% agar plates. Antibiotics were used at the following concentrations: erythromycin, 2.5 μg ml^-1^; tetracycline, 10 μg ml^-1^; and chloramphenicol, 2.5 μg ml^-1^. For the motility assay, strains were grown overnight in 2×YT liquid media at 28°C with shaking. One microliter of culture was then spotted onto the center of 2×YT plates. The overnight cultures generally contained an average of 8 × 10^7^ cells ml^-1^. For the motility assay, freshly prepared plates were allowed to dry overnight on the laboratory bench and used the next day. For the protein and RNA analyses, overnight cultures were diluted 10-fold, and 100 μl were spread on each 9 cm diameter plate. Cells were then cultivated at 37°C for 5 h prior to analysis. *Escherichia coli* HB101 was used for plasmid construction and was cultivated in LB medium. Ampicillin was used at 50 μg ml^-1^ when necessary. Plasmids were prepared using the FastGene Xpress Plasmid PLUS kit (Nippon Genetics) prior to electroporation of *Paenibacillus* sp. *Paenibacillus alvei* NBRC3343 was obtained from National Institute of Technology and Evaluation. *E*. *coli* W3100, *B*. *subtilis* NCIB 3610, and its *srfAC* mutant were described previously [29].

**Table 1:** *Paenibacillus* sp. strains used in this study.

Strains	Genotype	Source
NAIST15-1	Prototroph	This study
P155	Δ*fliF* (in frame deletion	pMADfliF → NAIST15-1
P261	Δ*hag* (in frame deletion)	pMADhag → NAIST15-1
P161	Δ*motAB*	pMADmotAB1→NAIST15-1
P158	Δ*motCD*	pMADmotAB2→NAIST15-1
P162	Δ*motAB*Δ*motCD*	pMADmotAB1→P158
P198	Δ*cmoA* (in frame deletion)	pMAD61 → NAIST15-1
P259	(the complemented strain of Δ*fliF*)	pMADfliFcomp → P155
P264	(the complemented strain of Δ*hag*)	pMADhagcomp → P261
P260	(the complemented strain of Δ*cmoA*)	pMAD61comp → P198
P200	Δ*PBN151_298—PBN151_299*	pMAD295-294 → NAIST15-1
P201	Δ*PBN151_1478—PBN151_1479*	pMAD713-714 → NAIST15-1
P101	Δ*hag*::*cat*	pMADhagC → NAIST15-1
P148	*hag* S161C	pMADhag161C → P101
P205	*cmoA-mCherry*	pMAD61RFP → NAIST15-1

### Microscopy

Colony formation was analyzed by time-lapse light microscopy. Briefly, 1 μl of *Paenibacillus* sp. suspension was spotted onto 2×YT/1.5% agar medium in a 35 mm diameter plate. The plate was then incubated at 37°C in a microscope incubation system (Tokai hit). The process of cell movement and colony formation was observed under a SZX7 zoom stereo microscope (Olympus) connected to a DP70 digital camera (Olympus). Cell movement was observed under a DMRE-HC microscope (Leica) connected to the DP70 digital camera. Time-lapse images and movies were collected using DP70 controller software (Olympus). Movies were downsized using Windows movie maker (Microsoft). Fluorescent images were observed under the DMRE-HC microscope connected to a digital CCD camera (1300Y; Roper Science). Image acquisition and processing were performed using Metamorph (Universal Imaging Corporation).

### Preparation of chromosomal DNA

*Paenibacillus* sp. was grown overnight in 5 ml of 2×YT at 28°C with shaking. Cells were then pelleted, suspended in 2 ml of saline-EDTA (150 mM NaCl, 50 mM EDTA, pH 8) supplemented with 0.5 mg ml^-1^ lysozyme, and incubated at 37°C for 20 min. Next, 1 μl of proteinase K (Takara Bio) was added and incubated at 37°C for 20 min. The lysate was then mixed with 200 μl of 10% SDS and incubated at 50°C until it became clear. Chromosomal DNA was extracted using phenol-chloroform-isoamyl alcohol and precipitated with ethanol. For draft genome sequencing, chromosomal DNA was purified using the Illustra bacteria genomic Prep Mini Spin Kit (GE Healthcare).

### Draft genome sequencing

Genomic DNA from *Paenibacillus* sp. was fragmented (500 bp fragments) using a sonicator (Covaris S2, Covaris). A library for sequencing was prepared using a NEBNext DNA Library Prep Master Mix Set for Illumina (New England Biolabs) and paired-end sequenced (2 × 300 bp) on a MiSeq sequencer using MiSeq reagent kit v. 3 (Illumina K.K.). Raw reads were trimmed and *de novo* assembled using CLC Genomics Workbench 6.5 (CLC bio, Qiagen).

### Nucleotide sequence accession numbers

The raw read data were deposited in the DRA database at DDBJ with the following accession numbers: DRA ID: DRA003610; BioProject ID: PRJDB3476; and BioSample ID: SAMD00030729. The draft genome sequence and annotation for *Paenibacillus* sp. NAIST15-1 were registered in DDBJ/EMBL/GenBank with the following accession numbers: BBYF01000001–BBYF01000042 (42 entries).

### Transformation of *Paenibacillus* sp.

*Paenibacillus* sp. was transformed by electroporation according to the method of Murray and Aronstein [66], with minor modifications. Briefly, *Paenibacillus* sp. was grown to OD_600_ = 0.2 in 2×YT at 37°C with shaking. The cells were then pelleted by centrifugation, washed three times with ice-cold 625 mM sucrose, and suspended in 1/200 culture volume of ice-cold 625 mM sucrose. Competent cells were aliquoted and stored at -80°C. Electroporation was performed using a Gene Pulser (Bio-Rad). For transformation, 45 μl of competent cells were mixed with 0.5–1 μg of plasmid DNA and transferred to an ice-cold 1 mm cuvette. The sample was then pulsed under the following conditions: voltage, 1.8 kV cm^−1^; capacitance, 25 μF; and resistance, 200 Ω. One milliliter of 2×YT was then added to the cell suspension and incubated overnight at 28°C with shaking. Transformants were selected on 2×YT/2.5% agar plates containing erythromycin (2.5 μg ml^-1^).

### Strain construction

Gene disruption was performed using the plasmid pMAD, which contains a thermosensitive origin of replication for Gram-positive bacteria, *erm*, and *bgaB*, which encodes a thermostable β-galactosidase [32]. pMAD was a kind gift from Dr. Kazuya Morikawa (Tsukuba University) and Dr. Michel Débarbouillé (Institut Pasteur). DNA fragments involved in gene disruption via double crossover recombination were prepared by PCR and cloned into pMAD. The plasmid pEpGAP-mCherry [67] was used to construct the *cmoA-mCherry* strain. pEpGAP-mCherry was a kind gift from Dr. Neta Dean (Stony Brook University). The primers used for PCR are listed in S1 Table. A detailed explanation of the methods used for plasmid construction and gene disruption are provided in S1 File.

### Fractionation of cellular proteins

Overnight cultures grown at 28°C in 2×YT were diluted 10-fold and 100 μl spread over 2×YT/1.5% agar plates. After 5 h of incubation at 37°C, cells from two plate cultures were suspended in 600 μl of 10 mM Tris-HCl (pH 7.6) and collected in 1.5 ml tubes. The volume of the samples was usually 150–200 μl. Cells were separated by centrifugation at 15,000 rpm for 2 min. The supernatant was made up to 200 μl with buffer and used as the secreted protein fraction. The precipitated cells were suspended in 200 μl of 10 mM Tris-HCl (pH 7.6)/0.1% SDS and boiled for 1.5 min to extract cell-surface associated proteins. The samples were then centrifuged at 15,000 rpm for 5 min. The supernatant was used as the cell-surface associated protein fraction. The precipitate was resuspended in 10 mM Tris-HCl (pH 7.6)/0.1% SDS and the cells disrupted by sonication. The sample was centrifuged at 15,000 rpm for 5 min and the supernatant was used as the cellular protein fraction. The three fractions were then separated by SDS-PAGE (SuperSep Ace 10–20% gels; Wako Pure Chemical Industries), and proteins were stained with Coomassie Brilliant Blue (CBB).

### LC-MS/MS analysis

After the cell-surface associated protein fraction was separated by SDS-PAGE, protein bands were stained with Coomassie Brilliant Blue and excised. Peptides were prepared for LC-MS/MS analysis by in-gel digestion with trypsin. LC-MS/MS analysis was performed using a LTQ-orbitrap XL system (Thermo Scientific), as described by Tanaka *et al*. [68]. The MS/MS spectra of the identified peptides were searched against our own database, which contains SpaA, flagellin, and CmoA sequences, and against non-redundant protein sequences in the National Center for Biotechnology Information (NCBI) database, using a MASCOT server (Matrix Science).

### Flagellar staining

Cells were collected from 2×YT liquid or plate cultures and pelleted by centrifugation at 5,000 rpm for 2 min. Cells were then suspended in T-base buffer [69]. Samples were mixed with Alexa Fluor 594 C5 maleimide (Life Technologies; final concentration, 15 μg ml^-1^) and incubated at room temperature for 1 h in the dark. The samples were then washed twice with T-base buffer and observed under a fluorescent microscope.

### Northern blot analysis

Cells were collected from 2×YT liquid or plate cultures. Total RNA was extracted and Northern blot analysis performed as described previously [70]. The primers used for RNA probe preparation are listed in S1 Table.

### Extracellular complementation

Overnight cultures grown at 28°C in 2×YT were diluted 10-fold and 100 μl spread over 2×YT/1.5% agar plates. After 5 h of incubation at 37°C, cells from two plate cultures were suspended in 800 μl of 2×YT liquid media and collected in 1.5 ml tubes. After brief vortexing, the sample was separated by centrifugation at 15,000 rpm for 5 min. The supernatant was sterilized by passing through a 0.45 μm PVDF membrane filter and 100 μl of the resultant sample was spread over a 1.5% agar plate. The plate was left for 10 min on the bench and used for the swarm assay.

## Supporting Information

S1 Fig *Paenibacillus* sp. produces swollen sporangia. *Paenibacillus* sp. was grown on 1.5% agar media at room temperature for 1 week. The cells were then observed by light microscopy. Many cells became swollen sporangia containing a phase-bright spore. Scale bar, 5 μm.
(TIF)

S2 Fig Pyrogenic tree of nucleotide sequences of the 16S rRNA gene fragments. Bacteria name and the GeneBank ID are shown. Pyrogenic tree was constructed using the web site, Phylogeny.fr (http://www.phylogeny.fr/).
(TIF)

S3 Fig Comparison of the *motAB* region of *Paenibacillus* sp. and the *swrA* region of *Bacillus subtilis*. Homologous genes are shown in the same color.
(TIF)

S4 Fig Putative pili in *Paenibacillus* sp. (A) Gene organization of putative pili genes of *Paenibacillus* sp. The deleting regions in strains Δ*PBN151_298-PBN151_299* and Δ*PBN151_1478-PBN151_1479* are shown above each map. (B) Motility of pili gene mutants on 2×YT/1.5% agar media.
(TIF)

S5 Fig Growth curve of *Paenibacillus* sp. mutants. Each strain was grown at 37°C in 2×YT liquid media. Strains, WT, wild-type; Δ*fliF*, P155; Δ*hag*, P261; Δ*motAB*, P168; Δ*motCD*, P158; Δ*motAB*Δ*motCD*, P162; and Δ*cmoA*, P198 shown in S1 Table.
(TIF)

S6 Fig Motility phenotype. Wild-type and mutant strains were inoculated onto the center of 2×YT/1.5% agar media and grown at 37°C for 18 h. Plate diameter, 9 cm. Strains: WT, wild-type; *fliF* complement, P259; *hag* complement, P264; *cmoA* complement, P260; *hag* S161C, P148; and *cmoA-mCherry*, P205 in S1 Table.
(TIF)

S7 Fig Flagellar stator proteins in *Paenibacillus* sp. (A) Comparison of MotAB and MotCD. Alignments of amino acid sequences of MotA vs MotC and MotB vs MotD are shown. Identical residues are shown by asterisks. (B) Phylogenetic tree of MotB stator proteins. Pyrogenic tree was constructed using the web site, Phylogeny.fr (http://www.phylogeny.fr/). This figure is based on Fig 4 of reference 55.
(TIF)

S8 Fig Fractionation of plate cultures of *Paenibacillus* sp. The wild-type strain was grown at 37°C for 5 h on 1.5% agar media. Secreted proteins (lane 1), cell-surface associated proteins (lane 2), and cellular proteins (lane 3) were extracted as described in Materials and Methods. The extracted proteins were then separated by SDS-PAGE. S-layer and flagellin proteins are indicated by arrows. The positions of molecular weight markers are shown.
(TIF)

S9 Fig LC-MS/MS analysis of S-layer (SpaA), flagellin (Hag), and CmoA. Peptides identified by a MASCOT search are indicated by red letters. Protein sequence coverage (%) is also shown.
(TIF)

S10 Fig Cell aggregates of motility-deficient mutants. The wild-type and mutant strains were spread over 2×YT/1.5% agar media and grown at 37°C.
(TIF)

S11 Fig Comparison of cell clusters between 0.5% and 0.6% agar media. The wild-type strain was grown at 37°C for 5 h on 0.5% or 0.6% agar media. Cells at the swarming edge region were observed. Scale bar, 20 μm.
(TIF)

S12 Fig Motility of *B*. *subtilis*, *P*. *alvei*, and *E*. *coli* on plates supplemented with CmoA. Overnight cultures of indicated bacterial strains were spotted on LB plates supplemented with the secreted protein fraction from the *Paenibacillus* sp. wild-type strain or the *cmoA* mutant. Plates were incubated at 37°C for 18 h. Strains, *B*. *subtilis* NCIB 3610, *B*. *subtilis* NCIB3610Δ*srfAC*, *Paenibacillus alvei* NBRC3343, and *E coli* W3100.
(TIF)

S13 Fig Isolation of bacteria exhibiting the colony scattering phenotype. Plant roots of weeds were soaked in 2 ml LB medium as described in Materials and Methods. After a brief vortex, the extract was incubated at 85°C for 10 min. Fifty μl of the extract was then plated onto LB/1.5% agar, and incubated at 37°C for 18 h. Several bacteria that exhibit the colony scattering phenotype are indicated by red arrows.
(TIF)

S1 Movie Small moving clusters of wild-type strain cells on a 1.5% agar plate. The wild-type strain was spotted on a 1.5% agar plate and incubated at 37°C for 6 h. Coverslips were placed directly onto the surface of the leading edge zones of the colonies and cell morphology observed under a video light microscope. The movie is real time. Scale bar, 20 μm.
(MP4)

S2 Movie Large moving clusters of wild-type strain cells on a 1.5% agar plate. The experiment was done as described in S1 Movie. The movie is real time. Scale bar, 20 μm.
(MP4)

S3 Movie Time-lapse analysis of colony formation. One microliter of *Paenibacillus* sp. suspension was spotted onto a 1.5% agar plate, which was then incubated at 37°C. The process of colony formation was analyzed by time-lapse light microscopy. Time-lapse images were collected every 1 min for 16 h after inoculation. Playback speed, ×1,440. Scale bar, 2 mm.
(MP4)

S4 Movie The *cmoA* mutant retains functional flagella. *cmoA* mutant cells were spread over the surface of a 1.5% agar plate and incubated at 37°C for 5 h. Two hundred microliters of water were poured onto the colonies and cellular behavior was immediately observed under a video light microscope. The movie is real time. Scale bar, 20 μm.
(MP4)

S5 Movie Cellular behavior of the wild-type strain at the leading edge zones of swarming colonies on 0.6% agar plates. The wild-type strain was spotted onto the center of 0.6% agar plates and incubated at 37°C for 6 h. Coverslips were placed directly on the surface of the leading edge zones of the colonies and cell morphology observed under a video light microscope. These movie is real time. Scale bar, 20 μm.
(MP4)

S6 Movie Cellular behavior of the *cmoA* mutant strain at the leading edge zones of swarming colonies on 0.6% agar plates. The experiment was done as described in S5 Movie. These movie is real time. Scale bar, 20 μm.
(MP4)

S7 Movie Wandering colonies are sensitive to wetness. Wild-type cells were spread over the surface of a 1.5% agar plate and incubated at 37°C until wandering colonies appeared. Two hundred microliters of water were poured onto the colonies and cellular behavior was immediately observed under a video light microscope. The movie is real time. Scale bar, 20 μm.
(MP4)

S1 Table Primers used in this study. (DOCX)

S1 File Supporting methods. (DOCX)

## References

[1] Snyder LA, Loman NJ, Fütterer K, Pallen MJ. Bacterial flagellar diversity and evolution: seek simplicity and distrust it? Trends Microbiol. 2009 1;17(1):1–5. Epub 2008 Dec 10. 10.1016/j.tim.2008.10.002 19081724

[2] Harshey RM. Bacterial motility on a surface: Many ways to a common goal. Annu Rev Microbiol 2003;57:249–73. 10.1146/annurev.micro.57.030502.091014 14527279

[3] Henrichsen J. Bacterial surface translocation: a survey and a classification. Bacteriol Rev. 1972 12;36(4):478–503. 4631369 10.1128/br.36.4.478-503.1972 PMC408329

[4] Jarrell KF, McBride MJ. 2008; The surprisingly diverse ways that prokaryotes move. Nat Rev Microbiol. 2008 6;6(6):466–76. 10.1038/nrmicro1900 18461074

[5] Kearns DB. A field guide to bacterial swarming motility. Nat Rev Microbiol. 2010 9;8(9):634–44. 10.1038/nrmicro2405 20694026 PMC3135019

[6] Partridge JD, Harshey RM. Swarming: flexible roaming plans. J Bacteriol. 2013 3;195(5):909–18. Epub 2012 Dec 21. 10.1128/JB.02063-12 23264580 PMC3571328

[7] Toguchi A, Siano M, Burkart M, Harshey RM. Genetics of swarming motility in Salmonella enterica serovar typhimurium: critical role for lipopolysaccharide. J Bacteriol. 2000 11;182(22):6308–21. 10.1128/jb.182.22.6308-6321.2000 11053374 PMC94776

[8] Wang Q, Suzuki A, Mariconda S, Porwollik S, Harshey RM. Sensing wetness: a new role for the bacterial flagellum. EMBO J. 2005 6 1;24(11):2034–42. Epub 2005 May 5. 10.1038/sj.emboj.7600668 15889148 PMC1142604

[9] Mariconda S, Wang Q, Harshey RM. A mechanical role for the chemotaxis system in swarming motility. Mol Microbiol. 2006 6;60(6):1590–602. 10.1111/j.1365-2958.2006.05208.x 16796690

[10] Wu Y, Berg HC. Water reservoir maintained by cell growth fuels the spreading of a bacterial swarm. Proc Natl Acad Sci U S A. 2012 3 13;109(11):4128–33. Epub 2012 Feb 27. 10.1073/pnas.1118238109 22371567 PMC3306679

[11] Turner L, Zhang R, Darnton NC, Berg HC. Visualization of Flagella during bacterial Swarming. J Bacteriol. 2010 7;192(13):3259–67. Epub 2010 Apr 2. 10.1128/JB.00083-10 20363932 PMC2897679

[12] López HM, Gachelin J, Douarche C, Auradou H, Clément E. Turning Bacteria Suspensions into Superfluids. Phys Rev Lett. 2015 7 10;115(2):028301 Epub 2015 Jul 7. 10.1103/PhysRevLett.115.028301 26207507

[13] Ash C, Priest FG, Collins MD. Molecular identification of rRNA group 3 bacilli (Ash, Farrow, Wallbanks and Collins) using a PCR probe test. Proposal for the creation of a new genus Paenibacillus. Antonie Van Leeuwenhoek. 1993–1994;64(3–4):253–60. 10.1007/BF00873085 8085788

[14] Genersch E, Forsgren E, Pentikäinen J, Ashiralieva A, Rauch S, Kilwinski J, et al Reclassification of Paenibacillus larvae subsp. pulvifaciens and Paenibacillus larvae subsp. larvae as Paenibacillus larvae without subspecies differentiation. Int J Syst Evol Microbiol. 2006 3;56(Pt 3):501–11. 10.1099/ijs.0.63928-0 16514018

[15] Lal S, Tabacchioni S. 2009; Ecology and biotechnological potential of Paenibacillus polymyxa: a minireview. Indian J Microbiol. 2009 3;49(1):2–10. Epub 2009 Apr 21. 10.1007/s12088-009-0008-y 23100748 PMC3450047

[16] McSpadden Gardener BB. Ecology of Bacillus and Paenibacillus spp. in Agricultural Systems. Phytopathology. 2004 11;94(11):1252–8. 10.1094/PHYTO.2004.94.11.1252 18944463

[17] Chou JH, Chou YJ, Lin KY, Sheu SY, Sheu DS, Arun AB, Young CC, Chen WM.Paenibacillus fonticola sp. nov., isolated from a warm spring. Int J Syst Evol Microbiol. 2007 6;57(Pt 6):1346–50. 10.1099/ijs.0.64872-0 17551056

[18] Roux V, Raoult D. Paenibacillus massiliensis sp. nov., Paenibacillus sanguinis sp. nov. and Paenibacillus timonensis sp. nov., isolated from blood cultures. Int J Syst Evol Microbiol. 2004 7;54(Pt 4):1049–54. 10.1099/ijs.0.02954-0 15280268

[19] Allard S, Enurah A, Strain E, Millner P, Rideout SL, Brown EW, et al In situ evaluation of Paenibacillus alvei in reducing carriage of Salmonella enterica serovar Newport on whole tomato plants. Appl Environ Microbiol. 2014 7;80(13):3842–9. Epub 2014 Apr 18. 10.1128/AEM.00835-14 24747888 PMC4054204

[20] Farag MA, Zhang H, Ryu CM. Dynamic chemical communication between plants and bacteria through airborne signals: induced resistance by bacterial volatiles. J Chem Ecol. 2013 7;39(7):1007–18. Epub 2013 Jul 24. 10.1007/s10886-013-0317-9 23881442 PMC3738840

[21] Sato I, Yoshida S, Iwamoto Y, Aino M, Hyakumachi M, Shimizu M, et al Suppressive potential of Paenibacillus strains isolated from the tomato phyllosphere against fusarium crown and root rot of tomato. Microbes Environ. 2014;29(2):168–77. Epub 2014 Jun 10. 10.1264/jsme2.me13172 24920171 PMC4103523

[22] Xie JB, Du Z, Bai L, Tian C, Zhang Y, Xie JY, et al Comparative genomic analysis of N2-fixing and non-N2-fixing Paenibacillus spp.: organization, evolution and expression of the nitrogen fixation genes. PLoS Genet. 2014 3 20;10(3):e1004231 eCollection 2014 Mar. 10.1371/journal.pgen.1004231 24651173 PMC3961195

[23] Cohen I, Ron IG, Ben-Jacob E. From branching to nebula patterning during colonial development of the Paenibacillus alvei bacteria. Pysica A 2000 286(1–2): 321–36. 10.1016/s0378-4371(00)00335-6

[24] Ingham CJ, Ben-Jacob E. Swarming and complex pattern formation in Paenibacillus vortex studied by imaging and tracking cells. BMC Microbiol. 2008 2 25;8:36 10.1186/1471-2180-8-36 18298829 PMC2268691

[25] Tcherpakov M, Ben-Jacob E, Gutnick DL. Paenibacillus dendritiformis sp. nov., proposal for a new pattern-forming species and its localization within a phylogenetic cluster. Int J Syst Bacteriol. 1999 1;49 Pt 1:239–46. 10.1099/00207713-49-1-239 10028268

[26] Hagai E, Dvora R, Havkin-Blank T, Zelinger E, Porat Z, Schulz S, et al Surface-motility induction, attraction and hitchhiking between bacterial species promote dispersal on solid surfaces. ISME J. 2014 5;8(5):1147–51 Epub 2013 Dec 5. 10.1038/ismej.2013.218 24304675 PMC3996695

[27] Ingham CJ, Kalisman O, Finkelshtein A, Ben-Jacob E. Mutually facilitated dispersal between the nonmotile fungus Aspergillus fumigatus and the swarming bacterium Paenibacillus vortex. Proc Natl Acad Sci U S A. 2011 12 6;108(49):19731–6 Epub 2011 Nov 21. 10.1073/pnas.1102097108 22106274 PMC3241745

[28] Be'er A, Strain SK, Hernández RA, Ben-Jacob E, Florin EL. Periodic reversals in Paenibacillus dendritiformis swarming. J Bacteriol. 2013 6;195(12):2709–17 Epub 2013 Apr 19 10.1128/JB.00080-13 23603739 PMC3697242

[29] Kobayashi K. Plant methyl salicylate induces defense responses in the rhizobacterium Bacillus subtilis. Environ Microbiol. 2015 4;17(4):1365–76. Epub 2014 Oct 7 10.1111/1462-2920.12613 25181478

[30] Kearns DB, Losick R. Cell population heterogeneity during growth of Bacillus subtilis. Genes Dev. 2005 12 15;19(24):3083–94 10.1101/gad.1373905 16357223 PMC1315410

[31] Mattick JS. Type IV pili and twitching motility. Annu Rev Microbiol. 2002;56:289–314. Epub 2002 Jan 30 10.1146/annurev.micro.56.012302.160938 12142488

[32] Arnaud M, Chastanet A, Débarbouillé M. New vector for efficient allelic replacement in naturally nontransformable, low-GC-content, gram-positive bacteria. Appl Environ Microbiol. 2004 11;70(11):6887–91. 10.1128/AEM.70.11.6887-6891.2004 15528558 PMC525206

[33] Minamino T, Imada K. The bacterial flagellar motor and its structural diversity. Trends Microbiol. 2015 5;23(5):267–74. 10.1016/j.tim.2014.12.011 25613993

[34] Ishiwa H., and Shibahara H. (1985) New shuttle vectors for *Escherichia coli* and *Bacillus subtilis*. II. Plasmid pHY300PLK, a multipurpose cloning vector with a polylinker, derived from pHY460. Jpn J Genet 60: 235–243. 10.1266/jjg.60.235

[35] Partridge JD, Harshey RM. More than motility: Salmonella flagella contribute to overriding friction and facilitating colony hydration during swarming. J Bacteriol. 2013 3;195(5):919–29. Epub 2012 Dec 21. 10.1128/JB.02064-12 23264575 PMC3571333

[36] Mukherjee S, Bree AC, Liu J, Patrick JE, Chien P, Kearns DB. Adaptor-mediated Lon proteolysis restricts Bacillus subtilis hyperflagellation. Proc Natl Acad Sci U S A. 2015 1 6;112(1):250–5. Epub 2014 Dec 23. 10.1073/pnas.1417419112 25538299 PMC4291670

[37] Tuson HH, Copeland MF, Carey S, Sacotte R, Weibel DB. Flagellum density regulates Proteus mirabilis swarmer cell motility in viscous environments. J Bacteriol. 2013 1;195(2):368–77. Epub 2012 Nov 9. 10.1128/JB.01537-12 23144253 PMC3553826

[38] van Ditmarsch D, Boyle KE, Sakhtah H, Oyler JE, Nadell CD, Déziel É, et al Convergent evolution of hyperswarming leads to impaired biofilm formation in pathogenic bacteria. Cell Rep. 2013 8 29;4(4):697–708. Epub 2013 Aug 15. 10.1016/j.celrep.2013.07.026 23954787 PMC3770465

[39] Zarschler K, Janesch B, Zayni S, Schäffer C, Messner P. Construction of a gene knockout system for application in Paenibacillus alvei CCM 2051T, exemplified by the S-layer glycan biosynthesis initiation enzyme WsfP. Appl Environ Microbiol. 2009 5;75(10):3077–85. Epub 2009 Mar 20. 10.1128/AEM.00087-09 19304819 PMC2681630

[40] Zarschler K, Janesch B, Pabst M, Altmann F, Messner P, Schäffer C. Protein tyrosine O-glycosylation—a rather unexplored prokaryotic glycosylation system. Glycobiology. 2010 6;20(6):787–98. Epub 2010 Mar 3. 10.1093/glycob/cwq035 20200052 PMC4397588

[41] Whittaker CA, Hynes RO. Distribution and evolution of von Willebrand/integrin A domains: widely dispersed domains with roles in cell adhesion and elsewhere. Mol Biol Cell. 2002 10;13(10):3369–87. 10.1091/mbc.E02-05-0259 12388743 PMC129952

[42] Bork P, Doerks T, Springer TA, Snel B. Domains in plexins: links to integrins and transcription factors. Trends Biochem Sci. 1999 7;24(7):261–3. 10.1016/s0968-0004(99)01416-4 10390613

[43] Janesch B, Koerdt A, Messner P, Schäffer C. The S-layer homology domain-containing protein SlhA from Paenibacillus alvei CCM 2051(T) is important for swarming and biofilm formation. PLoS One. 2013 9 18;8(9):e76566 eCollection 2013. 10.1371/journal.pone.0076566 24058714 PMC3776848

[44] Saha P, Mondal AK, Mayilraj S, Krishnamurthi S, Bhattacharya A, Chakrabarti T. Paenibacillus assamensis sp. nov., a novel bacterium isolated from a warm spring in Assam, India. Int J Syst Evol Microbiol. 2005 11;55(Pt 6):2577–81. 10.1099/ijs.0.63846-0 16280530

[45] Mead DA, Lucas S, Copeland A, Lapidus A, Cheng JF, Bruce DC, et al Complete genome sequence of Paenibacillus strain Y4.12MC10, a novel Paenibacillus lautus strain isolated from obsidian hot spring in Yellowstone national park. Stand Genomic Sci. 2012 7 30;6(3):381–400. Epub 2012 Jul 27. 10.4056/sigs.2605792 23408395 PMC3558958

[46] Chen L, Helmann JD. The Bacillus subtilis sigma D-dependent operon encoding the flagellar proteins FliD, FliS, and FliT. J Bacteriol. 1994 6;176(11):3093–101. 8195064 10.1128/jb.176.11.3093-3101.1994 PMC205476

[47] Copeland MF, Flickinger ST, Tuson HH, Weibel DB. Studying the dynamics of flagella in multicellular communities of Escherichia coli by using biarsenical dyes. Appl Environ Microbiol. 2010 2;76(4):1241–50 Epub 2009 Dec 18. 10.1128/AEM.02153-09 20023074 PMC2820973

[48] Be'er A, Harshey RM. Collective motion of surfactant-producing bacteria imparts superdiffusivity to their upper surface. Biophys J. 2011 9 7;101(5):1017–24. 10.1016/j.bpj.2011.07.019 21889437 PMC3164129

[49] Kearns DB, Losick R. Swarming motility in undomesticated Bacillus subtilis. Mol Microbiol. 2003 8;49(3):581–90. 12864845 10.1046/j.1365-2958.2003.03584.x

[50] Gode-Potratz CJ, Kustusch RJ, Breheny PJ, Weiss DS, McCarter LL. Surface sensing in Vibrio parahaemolyticus triggers a programme of gene expression that promotes colonization and virulence. Mol Microbiol. 2011 1;79(1):240–63. Epub 2010 Nov 16. 10.1111/j.1365-2958.2010.07445.x 21166906 PMC3075615

[51] Soo PC, Horng YT, Wei JR, Shu JC, Lu CC, Lai HC. Regulation of swarming motility and flhDC(Sm) expression by RssAB signaling in Serratia marcescens. J Bacteriol. 2008 4;190(7):2496–504. Epub 2008 Jan 25. 10.1128/JB.01670-07 18223092 PMC2293207

[52] Claret L, Hughes C. Rapid turnover of FlhD and FlhC, the flagellar regulon transcriptional activator proteins, during Proteus swarming. J Bacteriol. 2000 2;182(3):833–6. 10.1128/jb.182.3.833-836.2000 10633123 PMC94352

[53] McCarter L, Silverman M.Surface-induced swarmer cell differentiation of Vibrio parahaemolyticus. Mol Microbiol. 1990 7;4(7):1057–62. 10.1111/j.1365-2958.1990.tb00678.x 2233248

[54] Li N, Kojima S, Homma M. Sodium-driven motor of the polar flagellum in marine bacteria Vibrio. Genes Cells. 2011 10;16(10):985–99. Epub 2011 Sep 5. 10.1111/j.1365-2443.2011.01545.x 21895888

[55] Imazawa R, Takahashi Y, Aoki W, Sano M, Ito M.A novel type bacterial flagellar motor that can use divalent cations as a coupling ion. Sci Rep. 2016 1 22;6:19773 10.1038/srep19773 26794857 PMC4726428

[56] Lahaye E, Aubry T, Fleury V, Sire O.Does water activity rule P. mirabilis periodic swarming? II. Viscoelasticity and water balance during swarming. Biomacromolecules. 2007 4;8(4):1228–35. Epub 2007 Mar 14. 10.1021/bm070115w 17355121

[57] Ping L, Wu Y, Hosu BG, Tang JX, Berg HC. Osmotic pressure in a bacterial swarm. Biophys J. 2014 8 19;107(4):871–8. 10.1016/j.bpj.2014.05.052 25140422 PMC4142250

[58] Déziel E, Lépine F, Milot S, Villemur R. rhlA is required for the production of a novel biosurfactant promoting swarming motility in Pseudomonas aeruginosa: 3-(3-hydroxyalkanoyloxy)alkanoic acids (HAAs), the precursors of rhamnolipids. Microbiology. 2003 8;149(Pt 8):2005–13. 10.1099/mic.0.26154-0 12904540

[59] Jones BV, Young R, Mahenthiralingam E, Stickler DJ. Ultrastructure of Proteus mirabilis swarmer cell rafts and role of swarming in catheter-associated urinary tract infection. Infect Immun. 2004 7;72(7):3941–50. 10.1128/IAI.72.7.3941-3950.2004 15213138 PMC427392

[60] Chen G, Wang J, Xu X, Wu X, Piao R, Siu C.H. TgrC1 mediates cell-cell adhesion by interacting with TgrB1 via mutual IPT/TIG domains during development of Dictyostelium discoideum. Biochem J. 2013 6 1;452(2):259–69. 10.1042/BJ20121674 23477311

[61] Flemming HC, Wingender J. The biofilm matrix. Nat Rev Microbiol. 2010 9;8(9):623–33. Epub 2010 Aug 2. 10.1038/nrmicro2415 20676145

[62] Grau RR, de Oña P, Kunert M, Leñini C, Gallegos-Monterrosa R, Mhatre E, et al 2015; A duo of potassium-responsive histidine kinases govern the multicellular destiny of Bacillus subtilis. MBio. 2015 7 7;6(4):e00581 10.1128/mBio.00581-15 26152584 PMC4495169

[63] van Gestel J, Vlamakis H, Kolter R. From cell differentiation to cell collectives: Bacillus subtilis uses division of labor to migrate. PLoS Biol. 2015 4 20;13(4):e1002141 eCollection 2015 Apr. 10.1371/journal.pbio.1002141 25894589 PMC4403855

[64] Shi W, Zusman DR. The two motility systems of Myxococcus xanthus show different selective advantages on various surfaces. Proc Natl Acad Sci U S A. 1993 4 15;90(8):3378–82. 10.1073/pnas.90.8.3378 8475084 PMC46303

[65] Murray TS, Kazmierczak BI. Pseudomonas aeruginosa exhibits sliding motility in the absence of type IV pili and flagella. J Bacteriol. 2008 4;190(8):2700–8. Epub 2007 Dec 7. 10.1128/JB.01620-07 18065549 PMC2293233

[66] Murray KD, Aronstein KA. Transformation of the Gram-positive honey bee pathogen, Paenibacillus larvae, by electroporation. J Microbiol Methods. 2008 10;75(2):325–8. Epub 2008 Jul 17. 10.1016/j.mimet.2008.07.007 18687369

[67] Keppler-Ross S, Noffz C, Dean N. A new purple fluorescent color marker for genetic studies in Saccharomyces cerevisiae and Candida albicans. Genetics. 2008 5;179(1):705–10. 10.1534/genetics.108.087080 18493083 PMC2390648

[68] Tanaka N, Kato M, Tomioka R, Kurata R, Fukao Y, Aoyama T, et al Characteristics of a root hair-less line of Arabidopsis thaliana under physiological stresses. J Exp Bot. 2014 4;65(6):1497–512. Epub 2014 Feb 5. 10.1093/jxb/eru014 24501179 PMC3967087

[69] Blair KM, Turner L, Winkelman JT, Berg HC, Kearns DB. A molecular clutch disables flagella in the Bacillus subtilis biofilm. Science. 2008 6 20;320(5883):1636–8. 10.1126/science.1157877 18566286

[70] Kobayashi K. Gradual activation of the response regulator DegU controls serial expression of genes for flagellum formation and biofilm formation in Bacillus subtilis. Mol Microbiol. 2007 10;66(2):395–409. Epub 2007 Sep 10. 10.1111/j.1365-2958.2007.05923.x 17850253

