# Supplementary figures and images for: Genetic Analysis of Collective Motility of Paenibacillus sp. NAIST15-1
Source: PLoS Genet. 2016 Oct 20;12(10):e1006387. doi: 10.1371/journal.pgen.1006387 (PMC5072692; doi:10.1371/journal.pgen.1006387)

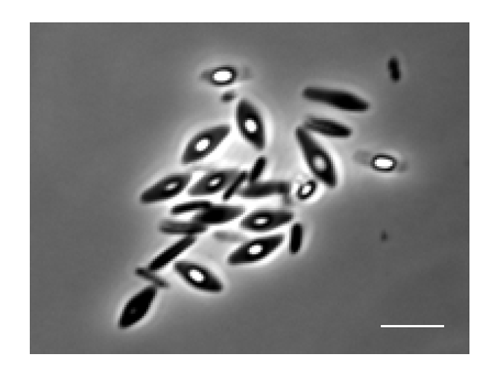

Supplement: S1 Fig — Paenibacillus sp. was grown on 1.5% agar media at room temperature for 1 week. The cells were then observed by light microscopy. Many cells became swollen sporangia containing a phase-bright spore. Scale bar, 5 μm. (TIF) [file pgen.1006387.s001.tif]

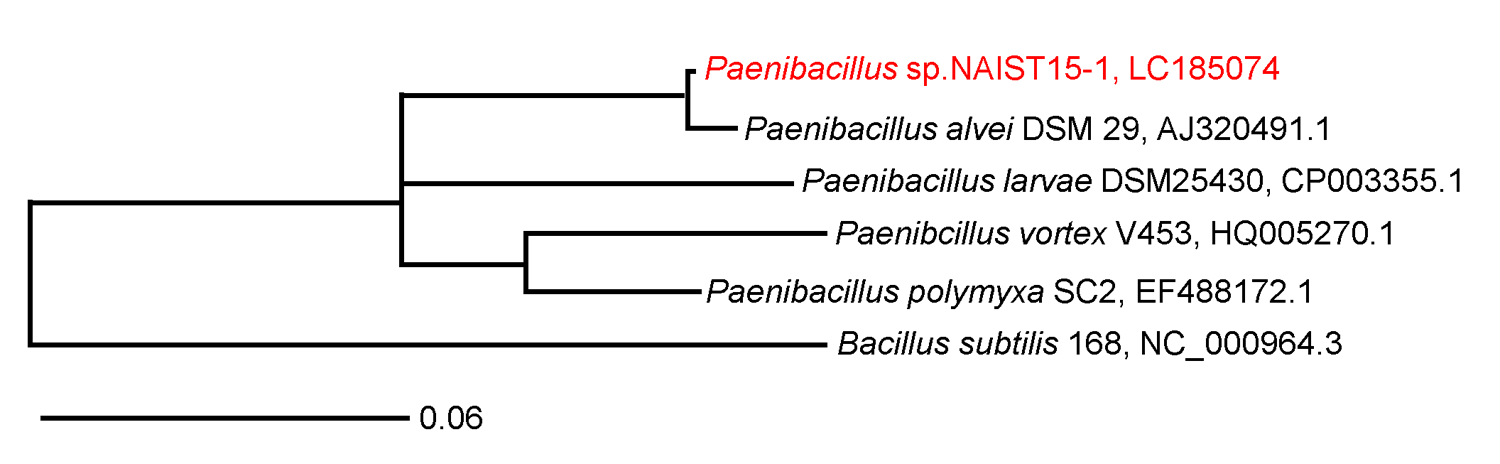

Supplement: S2 Fig — Bacteria name and the GeneBank ID are shown. Pyrogenic tree was constructed using the web site, Phylogeny.fr (http://www.phylogeny.fr/). (TIF) [file pgen.1006387.s002.tif]

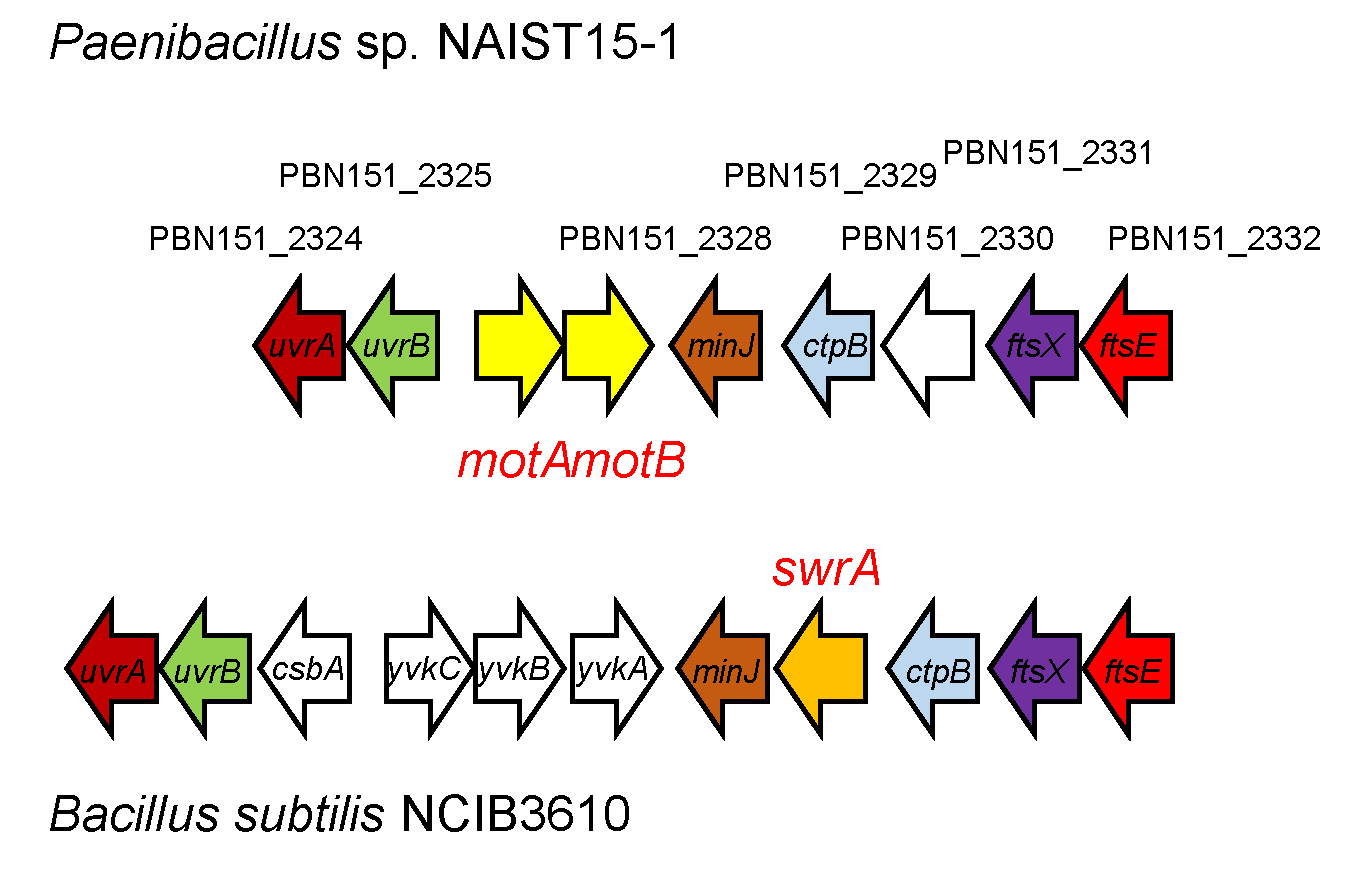

Supplement: S3 Fig — Homologous genes are shown in the same color. (TIF) [file pgen.1006387.s003.tif]

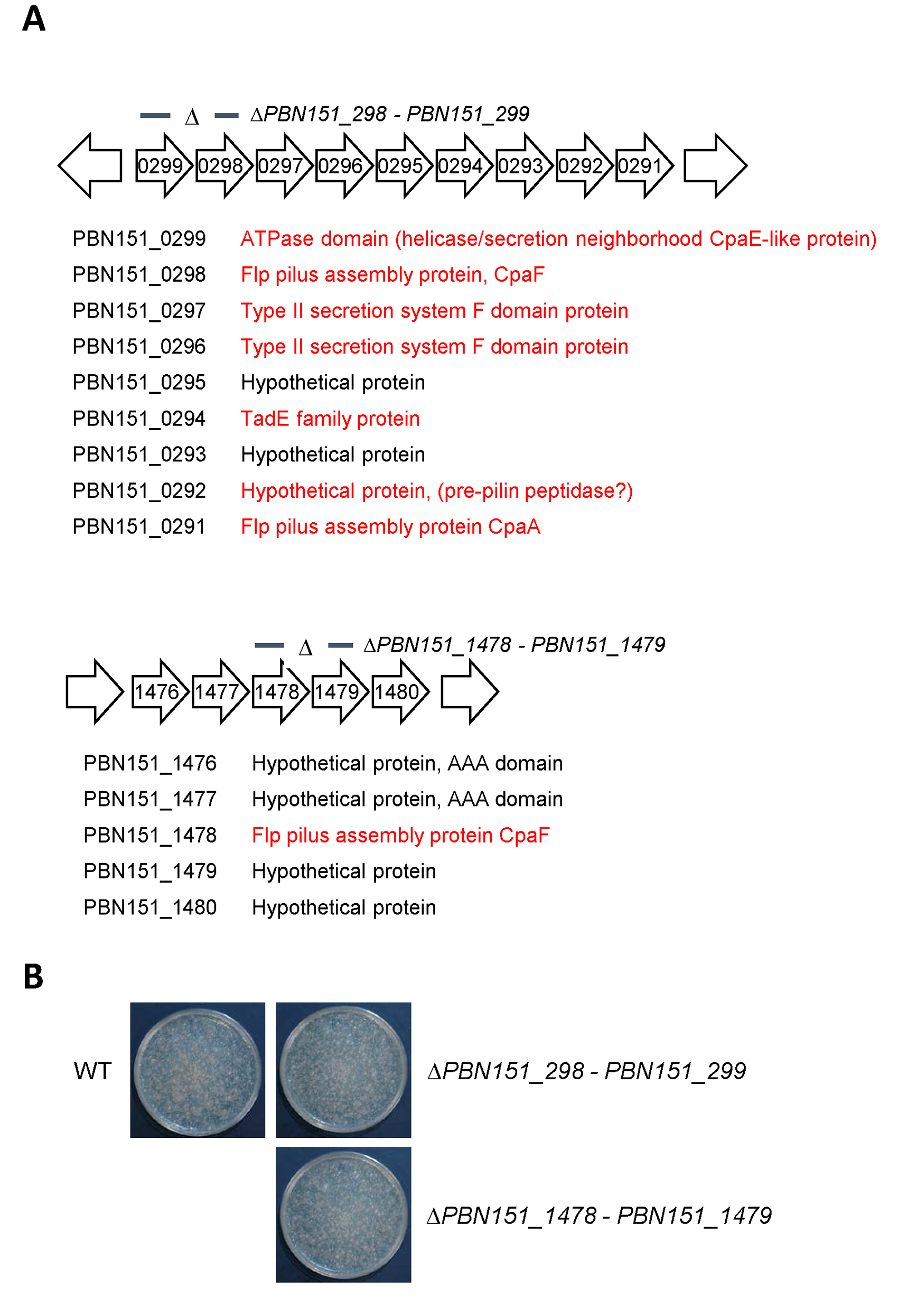

Supplement: S4 Fig — (A) Gene organization of putative pili genes of Paenibacillus sp. The deleting regions in strains ΔPBN151_298-PBN151_299 and ΔPBN151_1478-PBN151_1479 are shown above each map. (B) Motility of pili gene mutants on 2×YT/1.5% agar media. (TIF) [file pgen.1006387.s004.tif]

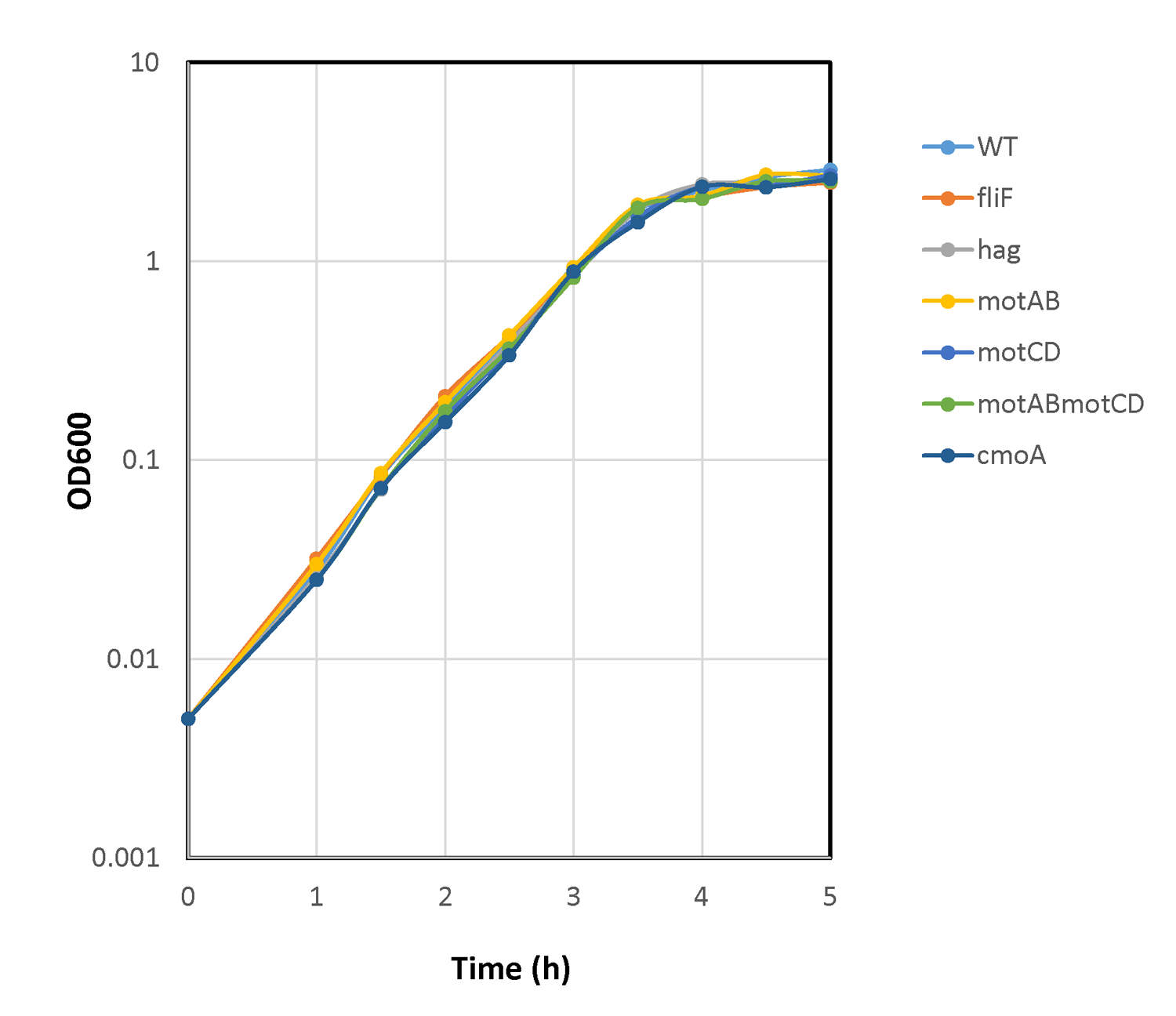

Supplement: S5 Fig — Each strain was grown at 37°C in 2×YT liquid media. Strains, WT, wild-type; ΔfliF, P155; Δhag, P261; ΔmotAB, P168; ΔmotCD, P158; ΔmotABΔmotCD, P162; and ΔcmoA, P198 shown in S1 Table. (TIF) [file pgen.1006387.s005.tif]

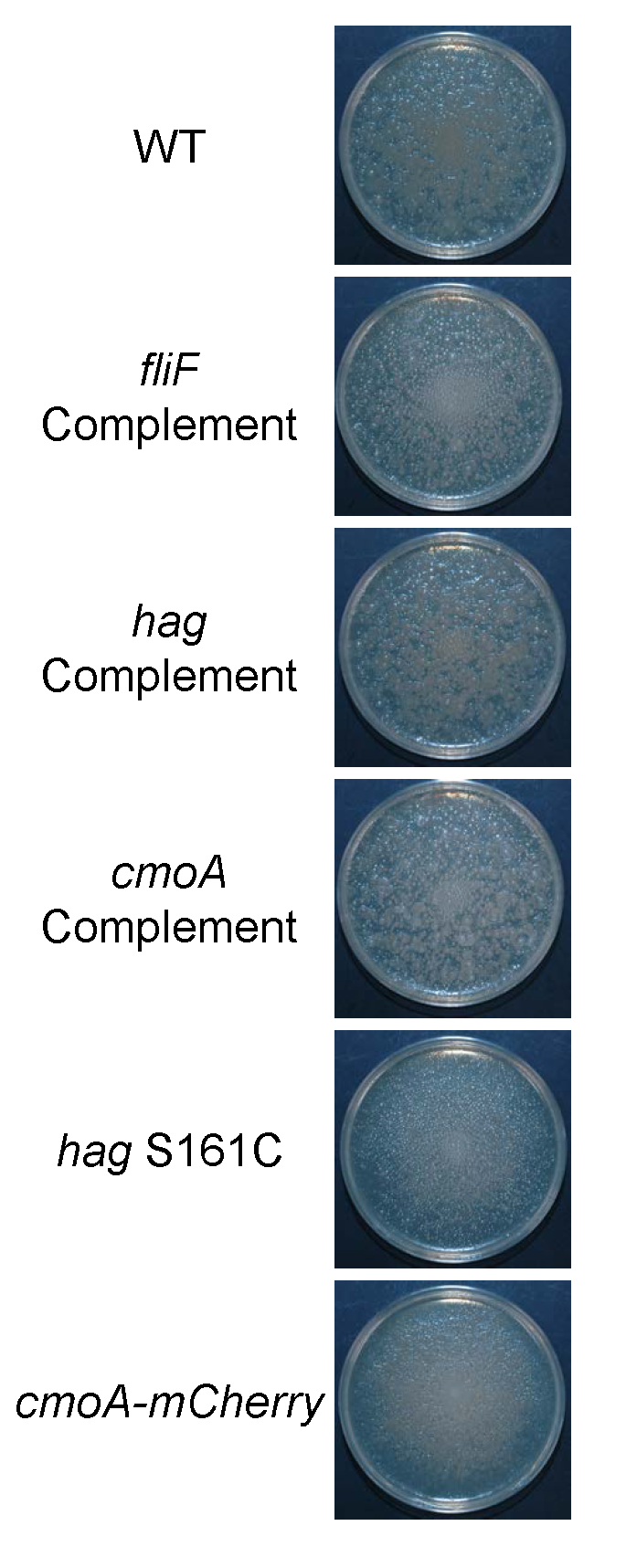

Supplement: S6 Fig — Wild-type and mutant strains were inoculated onto the center of 2×YT/1.5% agar media and grown at 37°C for 18 h. Plate diameter, 9 cm. Strains: WT, wild-type; fliF complement, P259; hag complement, P264; cmoA complement, P260; hag S161C, P148; and cmoA-mCherry, P205 in S1 Table. (TIF) [file pgen.1006387.s006.tif]

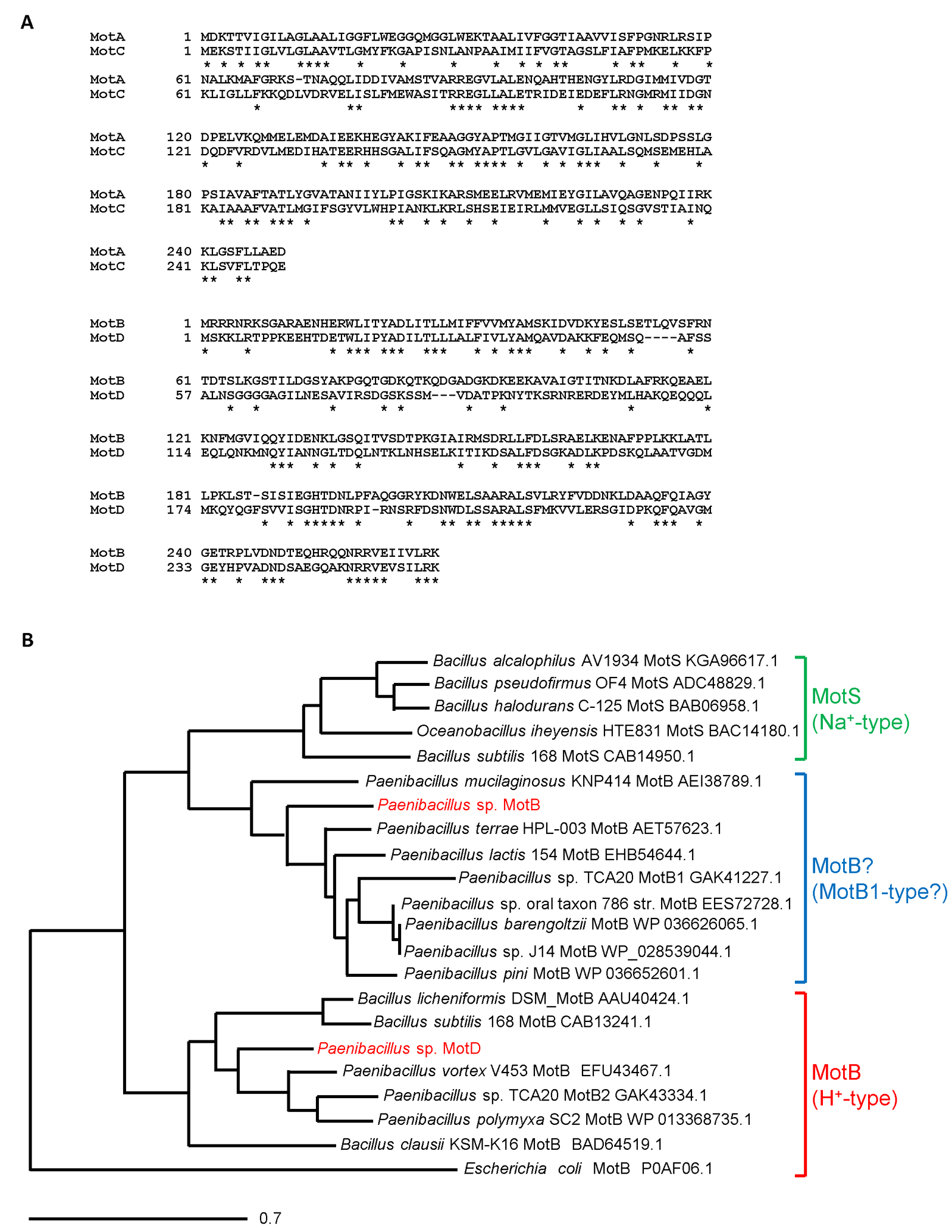

Supplement: S7 Fig — (A) Comparison of MotAB and MotCD. Alignments of amino acid sequences of MotA vs MotC and MotB vs MotD are shown. Identical residues are shown by asterisks. (B) Phylogenetic tree of MotB stator proteins. Pyrogenic tree was constructed using the web site, Phylogeny.fr (http://www.phylogeny.fr/). This figure is based on Fig 4 of reference 55. (TIF) [file pgen.1006387.s007.tif]

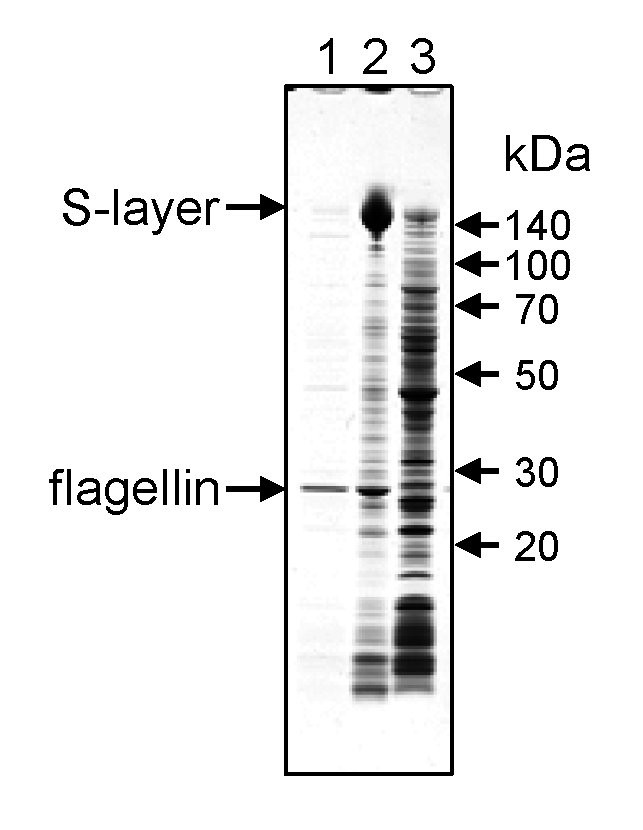

Supplement: S8 Fig — The wild-type strain was grown at 37°C for 5 h on 1.5% agar media. Secreted proteins (lane 1), cell-surface associated proteins (lane 2), and cellular proteins (lane 3) were extracted as described in Materials and Methods. The extracted proteins were then separated by SDS-PAGE. S-layer and flagellin proteins are indicated by arrows. The positions of molecular weight markers are shown. (TIF) [file pgen.1006387.s008.tif]

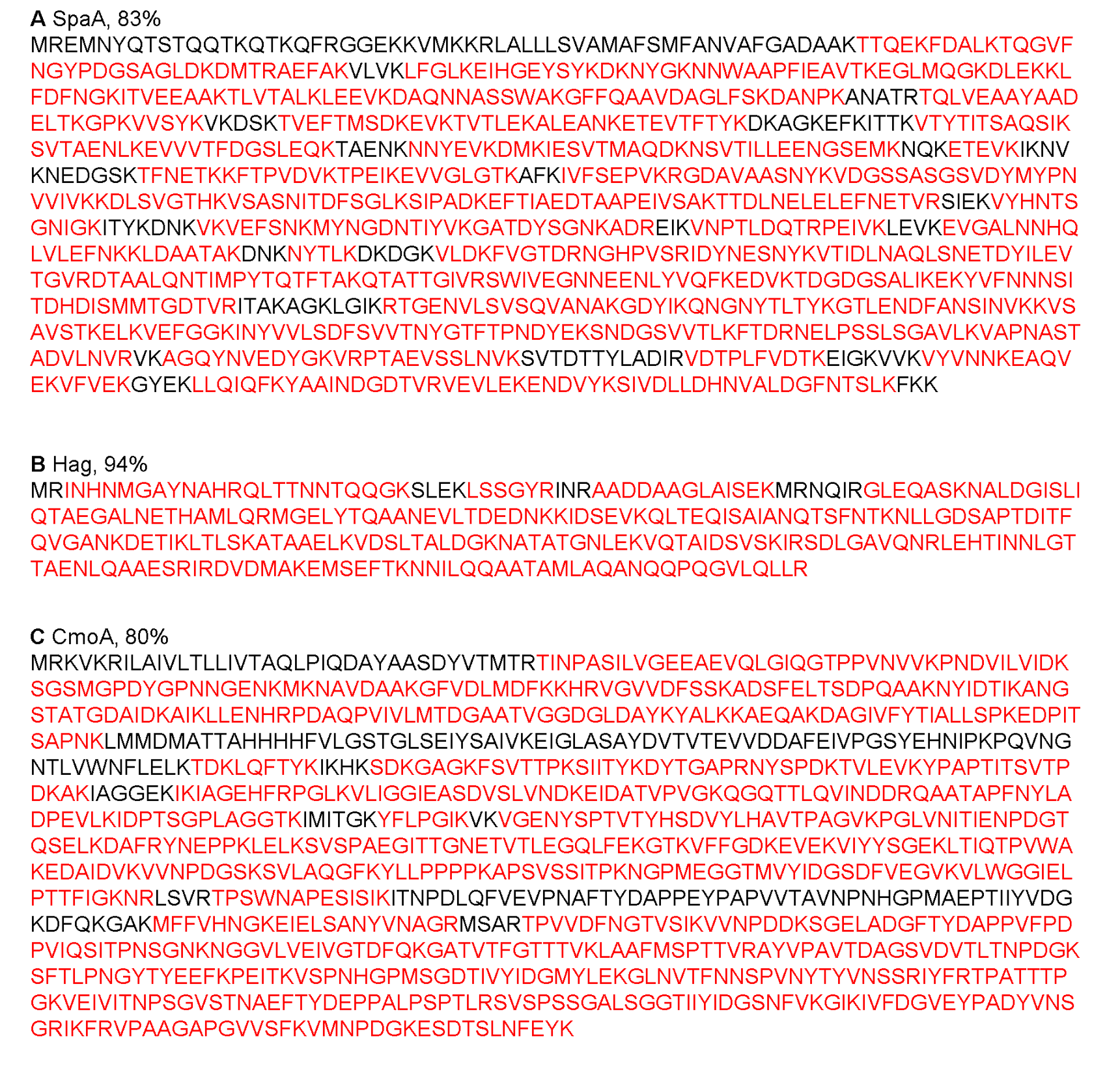

Supplement: S9 Fig — Peptides identified by a MASCOT search are indicated by red letters. Protein sequence coverage (%) is also shown. (TIF) [file pgen.1006387.s009.tif]

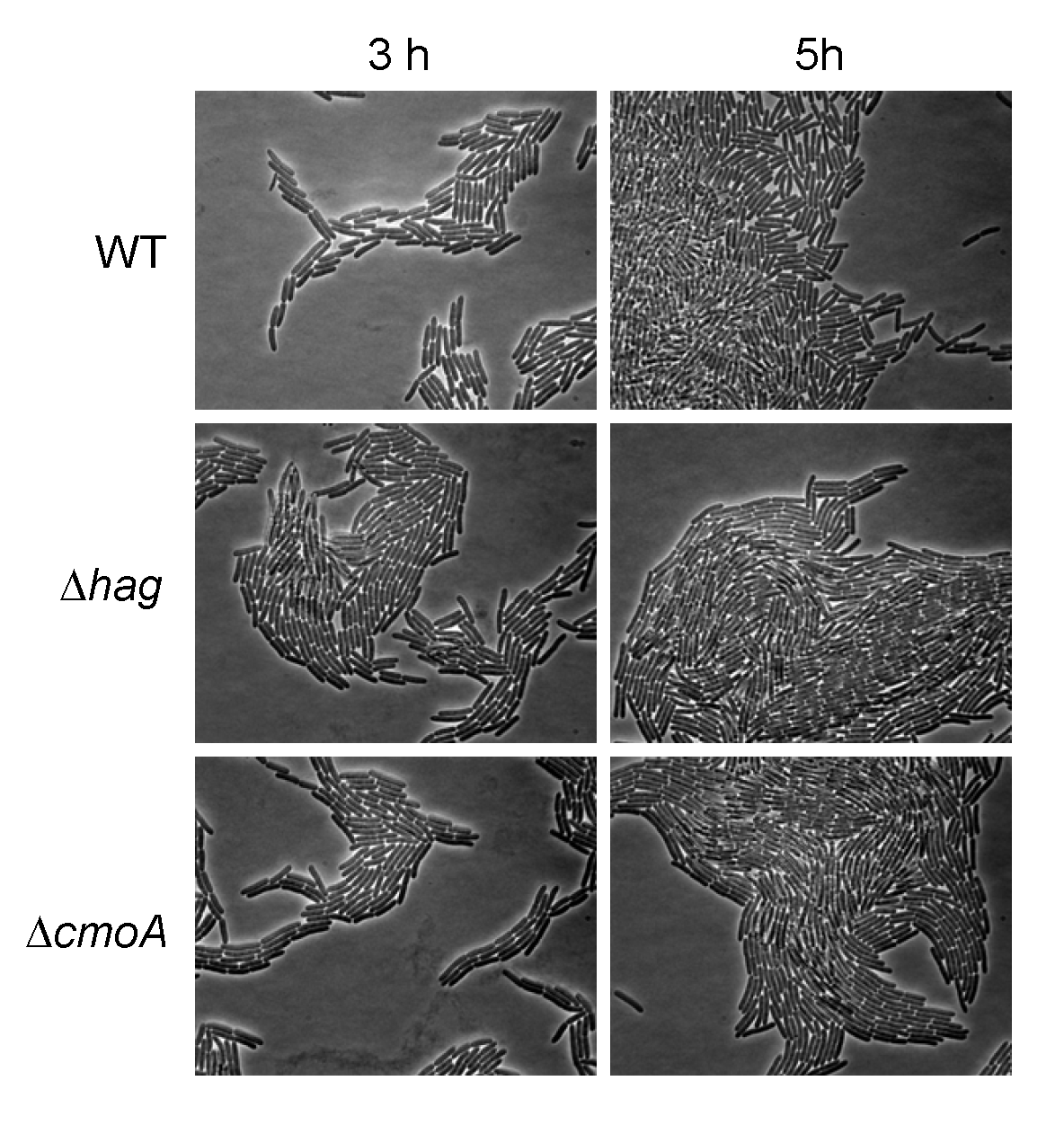

Supplement: S10 Fig — The wild-type and mutant strains were spread over 2×YT/1.5% agar media and grown at 37°C. (TIF) [file pgen.1006387.s010.tif]

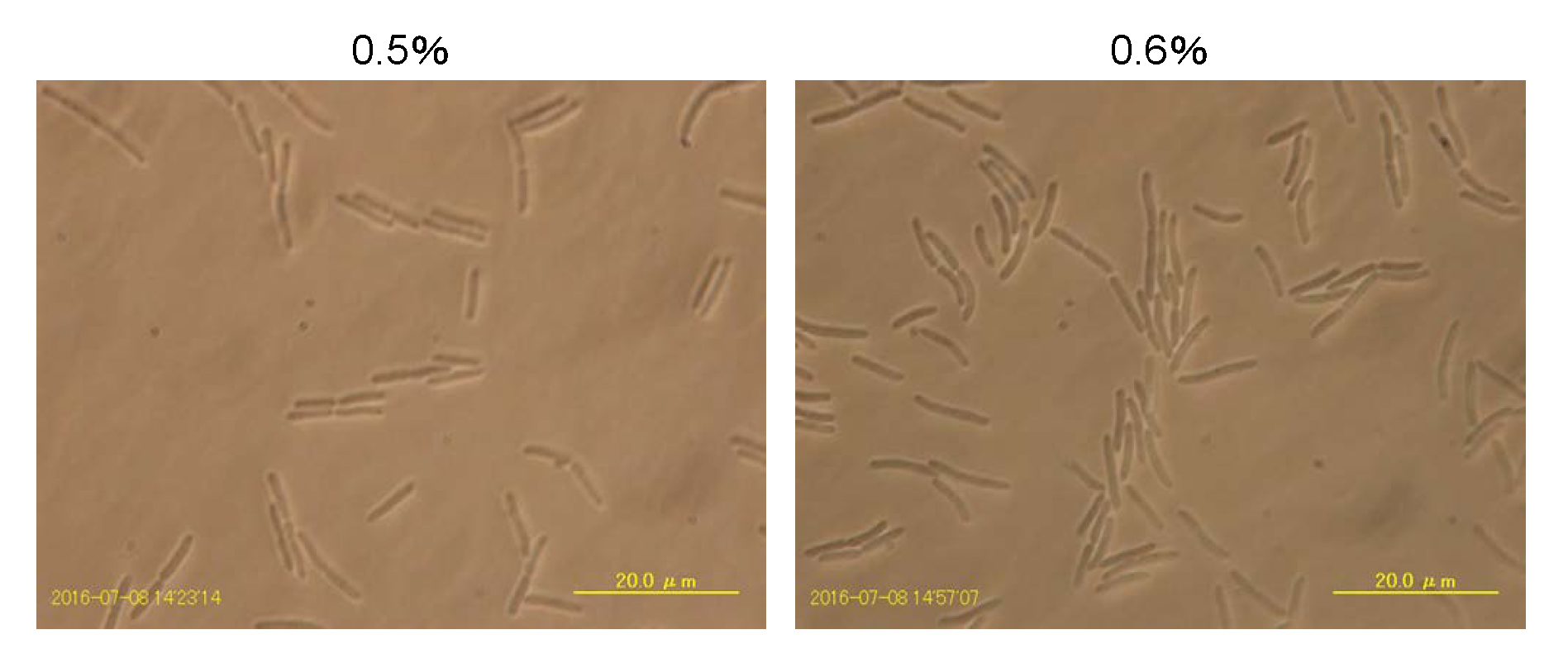

Supplement: S11 Fig — The wild-type strain was grown at 37°C for 5 h on 0.5% or 0.6% agar media. Cells at the swarming edge region were observed. Scale bar, 20 μm. (TIF) [file pgen.1006387.s011.tif]

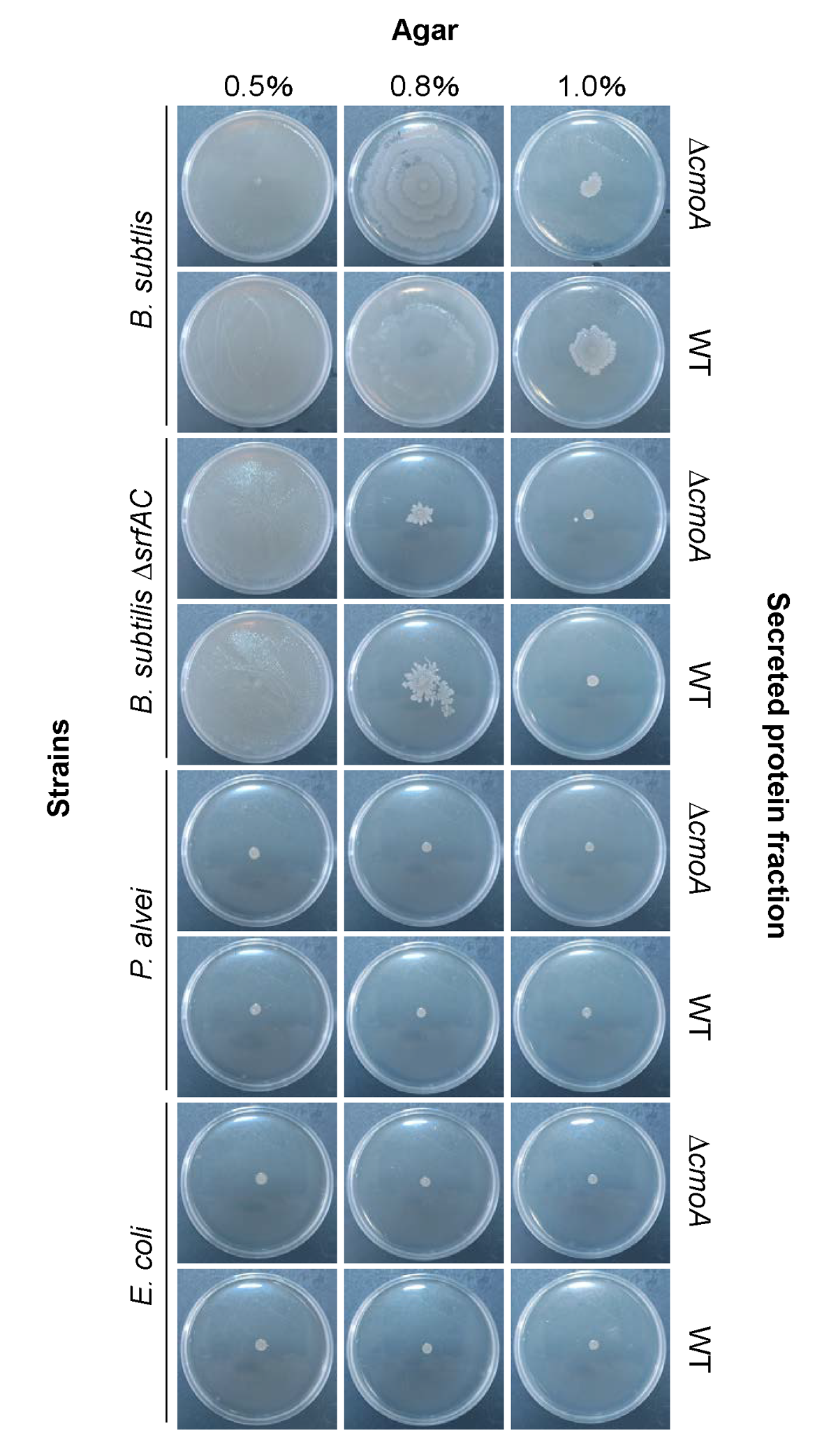

Supplement: S12 Fig — Overnight cultures of indicated bacterial strains were spotted on LB plates supplemented with the secreted protein fraction from the Paenibacillus sp. wild-type strain or the cmoA mutant. Plates were incubated at 37°C for 18 h. Strains, B. subtilis NCIB 3610, B. subtilis NCIB3610ΔsrfAC, Paenibacillus alvei NBRC3343, and E coli W3100. (TIF) [file pgen.1006387.s012.tif]

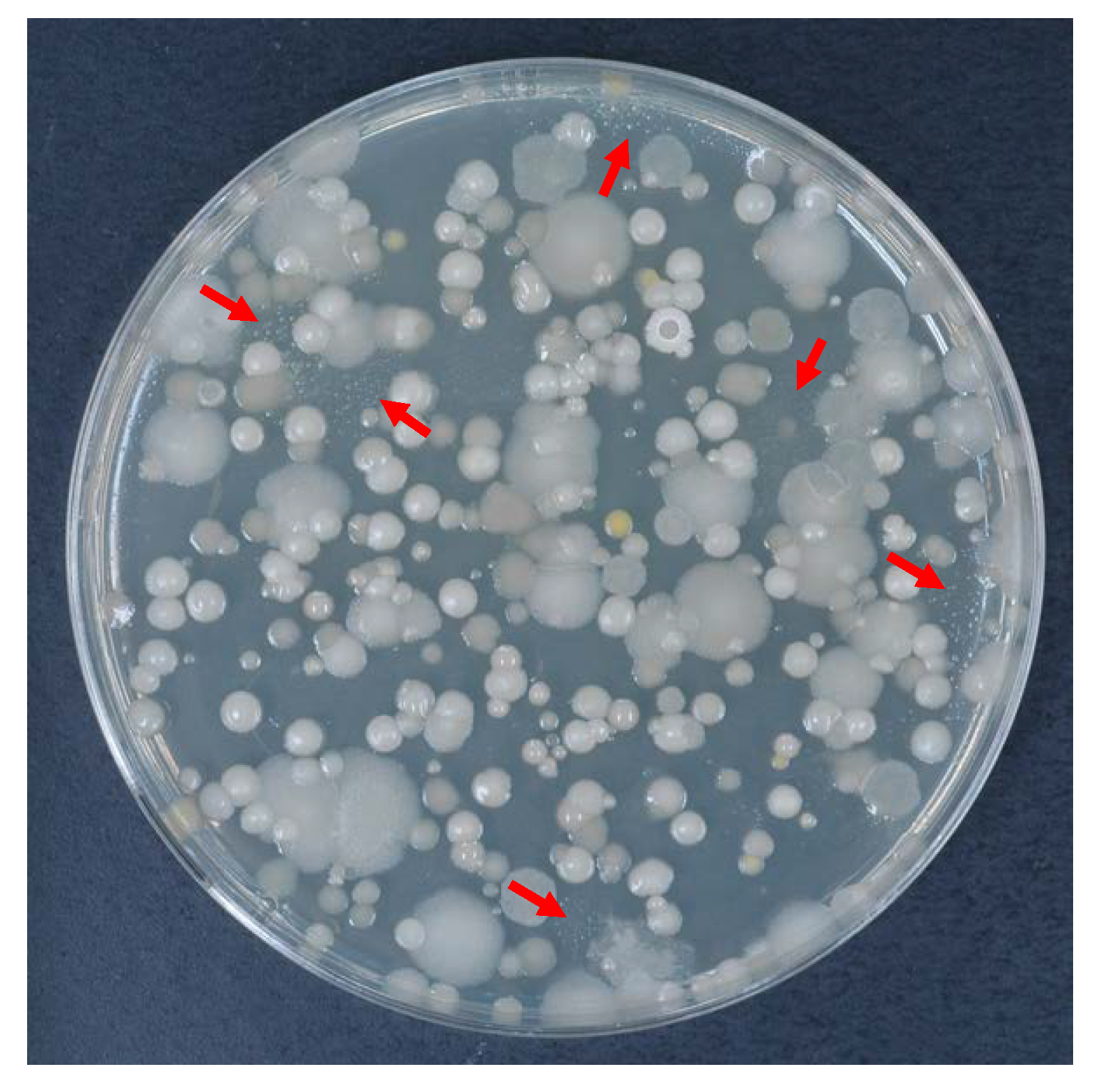

Supplement: S13 Fig — Plant roots of weeds were soaked in 2 ml LB medium as described in Materials and Methods. After a brief vortex, the extract was incubated at 85°C for 10 min. Fifty μl of the extract was then plated onto LB/1.5% agar, and incubated at 37°C for 18 h. Several bacteria that exhibit the colony scattering phenotype are indicated by red arrows. (TIF) [file pgen.1006387.s013.tif]
