# Supplementary material for: Genetic Analysis of Collective Motility of Paenibacillus sp. NAIST15-1
Source: PLoS Genet. 2016 Oct 20;12(10):e1006387. doi: 10.1371/journal.pgen.1006387 (PMC5072692; doi:10.1371/journal.pgen.1006387)
Supplement: S1 Table — (DOCX) [file pgen.1006387.s021.docx]

**S1 Table. Primers used in this study.**

| **Primers** | **Sequence (5' to 3')** |
| --- | --- |
| deletion mutants | |
| Δ*fliF* |  |
| fliF-D-F1 | TACGGCCGCAGCAGGGCTGAAGCAG |
| fliF-D-R1 | CAAATTCTCTAAGTCCACGATGGATGCCGATGCTTGTTCTTG |
| fliF-D-F2 | CATCGGCATCCATCGTGGACTTAGAGAATTTGACTGGCGAAC |
| fliF-D-R2 | AATTCCGGATCCTGAATTTCGAGAGAG |
| Δ*hag* | |
| hag-D-F1 | AAAGTCGTCGACGTGAAGTAGCTATATAGAACGCAAG |
| hag-D-R1-2 | TGTAGCAGCTTGTTGACGGTTAATACGGTAACCGGAAGAC |
| hag-D-F2-2 | TACCGTATTAACCGTCAACAAGCTGCTACAGCAATGCTGG |
| hag-D-R2 | GGAGGATCCTCATTACGGACTCGAGCGTAGAATC |
| Δ*motAB* | |
| motAB1-D-F1 | GGCTTCAGGCTGATAGTAATCGTAG |
| motAB1-D-R1 | GTTGTCCGTATGTCCACAGGAAGCCTCCGATGAGTGCAGC |
| motAB1-D-F2 | TCGGAGGCTTCCTGTGGACATACGGACAACCTACCGTTC |
| motAB1-D-R2 | AAAGAAGATCTGGCGTGTTGCTTGCTCCTGATGATC |
| Δ*motCD* | |
| motAB2-D-F1 | TCCTGCCAGTATCGCCATCAGGCAG |
| motAB2-D-R1 | CGGCTACAGGGTGGTAAGCCTTCGCGACGTGTAATCGACG |
| motAB2-D-F2 | ACGTCGCGAAGGCTTACCACCCTGTAGCCGACAATGATAG |
| motAB2-D-R2 | GGAGGATCCGCCGAATTGTTACGGACGTCGTCAC |
| Δ*cmoA* | |
| 61-D-F1 | GAAGAATTCAGCCAAGGTTCATGGTTAGCGCAAC |
| 61-D-R1 | ACTTACTACCGTCTGTGCATATGCATCCTGGATAGGGAGC |
| 61-D-F2 | CAGGATGCATATGCACAGACGGTAGTAAGTCAGTGCTCGC |
| 61-D-R2 | TCACAGGATCCGGAAATACAGGTGG |
| Δ*PBN151_298 - PBN151_299* | |
| 295-D-F1 | TTCATTCAGTGCCGAATCTGGTGTC |
| 295-D-R1 | TTTCGCTGTCTTTACAGCTCCCGAATCATATCCACATCAG |
| 294-D-F2 | TATGATTCGGGAGCTGTAAAGACAGCGAAATGGCTTCCAG |
| 294-D-R2 | AAAGAAGATCTATTGCTTATTCCTCACCCTTGCATC |
| Δ*PBN151_1478 - PBN151_1479* | |
| 713-D-F1 | GCAGCAATACGACGGAGATGAGGAG |
| 713-D-R1 | TGTCTAAGCATATCCGTTTCAGAGCGGTTATATCCTGCTC |
| 714-D-F2 | TAACCGCTCTGAAACGGATATGCTTAGACAGCATTATGAG |
| 714-D-R2 | GGAGGATCCACAGTATCGATGCTACTGCTAGTTC |
| *cat* insertion mutant | |
| cat-F | TCTTCAACTAAAGCACCCATTAG |
| cat-R | AGTACAGTCGGCATTATCTC |
| Δ*hag*::*cat* | |
| hag-D-F1 | AAAGTCGTCGACGTGAAGTAGCTATATAGAACGCAAG |
| hag-D-R1 | CTAATGGGTGCTTTAGTTGAAGAACGGTTAATACGGTAACCGGAAGAC |
| hag-D-F2 | TATGAGATAATGCCGACTGTACTTCAACAAGCTGCTACAGCAATGCTG |
| hag-D-R2 | GGAGGATCCTCATTACGGACTCGAGCGTAGAATC |
| *other mutants* | |
| *hag* S161C | |
| hag-D-F1(Sal) | AAAGTCGTCGACGTGAAGTAGCTATATAGAACGCAAG |
| Cys-F2 | CTATCAAGTTGACATTGTGCAAAGCAACTGCTGCTGA |
| Cys-R2 | TCAGCAGCAGTTGCTTTGCACAATGTCAACTTGATAG |
| hag-D-R2 | GGAGGATCCTCATTACGGACTCGAGCGTAGAATC |
| *cmoA-mCherry* | |
| 60-D-F1 | GTTTACTCTACCAAACGGATATACG |
| 61-mCherry-R1 | CTTCTTCACCTTTTGAGTACTCAAAGTTTAATGAAGTATC |
| 61-mCherry-F2 | AATTATATAAATAATGAGTACAAATAATGGGAAAGGGATCTTC |
| 61-GFPtc-R2 | AAAGAAGATCTGCATAATCAATCGATCGCTAATCGC-- |
| 61-mCherry-F1 | TAAACTTTGAGTACTCAAAAGGTGAAGAAGATAATATGGC |
| 61-mCherry-R2 | CCATTATTTGTACTCATTATTTATATAATTCATCCATACC |
| Probes for Northern blot analysis | |
| hag-N-F | GAAGAATTCGCGTATTAACCACAACATGGGTGCC |
| hag-N-T7R | TAATACGACTCACTATAGGGCGAGCCAGCATTGCTGTAGCAGC |
| motAB-N-F | TTAGCTGCACTCATCGGAGG |
| motAB-N-T7R | TAATACGACTCACTATAGGGCGAATCATGAGCAGGGTGATGAG |
| motCD-N-F | GAGTGGGCGTCGATTACACG |
| motCD-N-T7R | TAATACGACTCACTATAGGGCGACCATCTGATCGAATGACTGC |
| 61-D-F2 | CAGGATGCATATGCACAGACGGTAGTAAGTCAGTGCTCGC |
| 60-N-T7R | TAATACGACTCACTATAGGGCGACGTATATCCGTTTGGTAGAG |
