# Supplementary material for: Genetic Analysis of Collective Motility of Paenibacillus sp. NAIST15-1
Source: PLoS Genet. 2016 Oct 20;12(10):e1006387. doi: 10.1371/journal.pgen.1006387 (PMC5072692; doi:10.1371/journal.pgen.1006387)
Supplement: S1 File — (DOCX) [file pgen.1006387.s022.docx]

**Supporting information**

**Construction of deletion mutants**

To construct the deletion mutants, the regions upstream and downstream of the target genes were PCR amplified from *Paenibacillus* sp. chromosomal DNA using gene-specific primer pairs D-F1/D-R1 and D-F2/D-R2, respectively. The primer sequences are listed in S1 Table. The 5′ sequence of D-R1 was complementary to the 5’ sequence of D-F2. The two PCR fragments were extended and connected by mixing them together and using them as a template for a second PCR amplification using primers D-F1 and D-R2. The resulting PCR products were then digested with restriction enzymes and cloned into the pMAD plasmid (Arnaud *et al*, 2004).

To construct the *cat* insertion mutants, the regions upstream and downstream of the target genes were PCR amplified from chromosomal DNA using gene-specific primer pairs D-F1/D-R1 and D-F2/D-R2. The 5′ D-R1 and D-F2 sequences were complementary to the cat-F and cat-R sequences, respectively. A *cat* cassette was PCR amplified from pCBB31 (Kobayashi, 2007) using primers cat-F and cat-R. The three PCR fragments were extended and connected by mixing them together and using them as a template for a second PCR amplification using primers D-F1 and D-R2. The resulting PCR products were then digested with restriction enzymes and cloned into the pMAD plasmid.

The pMAD-based plasmids were introduced into *Paenibacillus* sp. by electroporation and transformants selected by growth at 30°C on 2×YT/2.5% agar supplemented with 2.5 μg ml^-1^ erythromycin (Erm) and 100 μg ml^-1^ 5-bromo-4-chloro-indol-β-D-galactopyranoside (X-gal). Fresh Erm^r^ colonies were picked, spread on 2×YT-Erm-X-gal/2.5% agar plates, and integrants (harboring plasmids inserted into the chromosome via a single crossover recombination) selected at 43°C. Erm^r^ blue colonies were picked and cultivated overnight in 2×YT at 28°C with shaking. The cultures were then diluted 10-fold with 2×YT and cultivated at 30°C for 2.5 h. The cultures were again diluted 10-fold and cultivated at 43°C for 3 h. Finally, the cultures were spread on 2×YT-X-gal/2.5% agar plates with appropriate dilution and incubated at 43°C. White colonies (resulting from loss of the plasmid) were picked out and chromosomal DNA prepared. Gene disruption mutants (resulting from a double crossover recombination) were then identified by PCR.

For the complementation test, the regions including the target genes were PCR amplified from *Paenibacillus* sp. chromosomal DNA using gene-specific primer pairs D-F1/D-R2, which were also used for mutant construction. The resulting PCR products were then digested with restriction enzymes and cloned into the pMAD plasmid. The resultant plasmids were introduced into the mutant strains, and complementation strains were isolated using procedures identical to those used to isolate the disruption mutants.

**Construction of the *hag* S161C mutant**

The 5’ and 3’ regions of *hag* were PCR amplified from *Paenibacillus* sp. chromosomal DNA using primer pairs hag-D-F1(Sal)/CysR2 and Cys-F2/hag-D-R2, respectively. The 5’ sequences of CysR2 and Cys F2 were complementary to each other and contained a TCA (Ser) to TGC (Cys) substitution. To extend and connect the two PCR fragments, the two fragments were mixed and used as a template for a second PCR amplification using primers hag-D-F1 and hag-D-R2. The resulting PCR products were digested with restriction enzymes and cloned into the pMAD plasmid. The same procedures were used to isolate the *hag* S161C mutant, except that the resulting plasmid was introduced into the Δ*hag*::*cat* mutant. The *hag* S161C mutant was identified from among the white Cm^s^ colonies by PCR.

**Construction of the *cmoA-mCherry* strain**

The 3’ part of *cmoA* (lacking the stop) codon and the downstream region of cmoA were PCR amplified from *Paenibacillus* sp. chromosomal DNA using primer pairs 60-D-F1/61-mCherry-R1 and 60-mCherry-F2/61-GFPtc-R2, respectively. The DNA region of *mCherry* was amplified from the plasmid pEpGAP (Keppler-Ross *et al*., 2008) using primers 61-mCherry-F1/61-mCherry-R2. The 5’ sequences of 61-mCherry-R1 and 61-mCherry–F2 were complementary to the 5’ sequence of 61-mCherry-F1 and 61-mCherry-R2, respectively. The three PCR fragments were extended and connected by mixing them together and using them as a template for a second PCR amplification using primers 60-D-F1 and 61-GFPtc-R2. The resulting PCR products were digested with restriction enzymes and cloned into the pMAD plasmid. The resulting plasmid was introduced into the wild-type strain and the *cmoA-mCherry* strain was isolated as described above.

**Construction of pHYmotAB and pHYmotCD.**

DNA regions of *motAB* and *motCD* operons were amplified with primer pairs, motAB1-D-F1/motAB1-D-R2 and motAB2-D-F1/motAB2-D-R2, respectively. Those primers are the same primers used for gene disruption. After digestion with restriction enzymes, those DNA fragments were cloned in the multicopy plasmid pHY300PLK (Ishiwa *et al*., 1985).

**References for Supporting information**

Arnaud M, Chastanet A, Débarbouillé M. New vector for efficient allelic replacement in naturally nontransformable, low-GC-content, gram-positive bacteria. Appl Environ Microbiol. 2004 Nov;70(11):6887-91. PMID: 15528558.

Keppler-Ross S, Noffz C, Dean N. A new purple fluorescent color marker for genetic studies in Saccharomyces cerevisiae and *Candida albicans*. Genetics. 2008 May;179(1):705-10. doi: 10.1534/genetics.108.087080. PMID: 18493083

Kobayashi K. *Bacillus subtilis* pellicle formation proceeds through genetically defined morphological changes. J Bacteriol. 2007 Jul;189(13):4920-31. Epub 2007 Apr 27. PMID: 17468240.

*Ishiwa, H., and Shibahara.* H. New shuttle vectors for *Escherichia coli* and *Bacillus subtilis*. II. Plasmid pHY300PLK, a multipurpose cloning vector with a polylinker, derived from pHY460. *Jpn J Genet* 60: 235-43.
